# Oligodendrocyte Precursor Cells Modulate the Neuronal Network by Activity-Dependent Ectodomain Cleavage of Glial NG2

**DOI:** 10.1371/journal.pbio.1001993

**Published:** 2014-11-11

**Authors:** Dominik Sakry, Angela Neitz, Jeet Singh, Renato Frischknecht, Daniele Marongiu, Fabien Binamé, Sumudhu S. Perera, Kristina Endres, Beat Lutz, Konstantin Radyushkin, Jacqueline Trotter, Thomas Mittmann

**Affiliations:** 1 Molecular Cell Biology, Department of Biology, Johannes Gutenberg University Mainz, Mainz, Germany; 2 Institute of Physiology, University Medical Center of the Johannes-Gutenberg University Mainz, Mainz, Germany; 3 Leibniz Institute for Neurobiology, Magdeburg, Germany; 4 Department of Psychiatry and Psychotherapy, University Medicine Mainz, Mainz, Germany; 5 Institute of Physiological Chemistry, University Medical Center Mainz, Mainz, Germany; 6 Mouse Behavior Outcome Unit, Focus Program Translational Neurosciences (FTN), Johannes Gutenberg University Mainz, Mainz, Germany; Stanford University School of Medicine, United States of America

## Abstract

The role of glia in modulating neuronal network activity is an important question. Oligodendrocyte precursor cells (OPC) characteristically express the transmembrane proteoglycan nerve-glia antigen 2 (NG2) and are unique glial cells receiving synaptic input from neurons. The development of NG2+ OPC into myelinating oligodendrocytes has been well studied, yet the retention of a large population of synapse-bearing OPC in the adult brain poses the question as to additional functional roles of OPC in the neuronal network. Here we report that activity-dependent processing of NG2 by OPC-expressed secretases functionally regulates the neuronal network. NG2 cleavage by the α-secretase ADAM10 yields an ectodomain present in the extracellular matrix and a C-terminal fragment that is subsequently further processed by the γ-secretase to release an intracellular domain. ADAM10-dependent NG2 ectodomain cleavage and release (shedding) in acute brain slices or isolated OPC is increased by distinct activity-increasing stimuli. Lack of NG2 expression in OPC (NG2-knockout mice), or pharmacological inhibition of NG2 ectodomain shedding in wild-type OPC, results in a striking reduction of N-methyl-D-aspartate (NMDA) receptor-dependent long-term potentiation (LTP) in pyramidal neurons of the somatosensory cortex and alterations in the subunit composition of their α-amino-3-hydroxy-5-methyl-4-isoxazolepr opionicacid (AMPA) receptors. In NG2-knockout mice these neurons exhibit diminished AMPA and NMDA receptor-dependent current amplitudes; strikingly AMPA receptor currents can be rescued by application of conserved LNS protein domains of the NG2 ectodomain. Furthermore, NG2-knockout mice exhibit altered behavior in tests measuring sensorimotor function. These results demonstrate for the first time a bidirectional cross-talk between OPC and the surrounding neuronal network and demonstrate a novel physiological role for OPC in regulating information processing at neuronal synapses.

## Abstract 2

This study shows that the activity of neurons can trigger shedding of a protein, NG2, from the surface of oligodendrocyte precursor cells; this protein in turn modulates synaptic transmission, revealing a two-way conversation between neurons and glia.

## Author Summary

Although glial cells substantially outnumber neurons in the mammalian brain, much remains to be discovered regarding their functions. Among glial cells, oligodendrocyte precursors differentiate into oligodendrocytes, whose function is to enwrap nerves with myelin to ensure proper impulse conduction. However, oligodendrocyte precursors also comprise a stable population in all major regions of the adult brain, making up around 5% of the total number of neurons and glia. Synapses are classically formed between neurons. Nonetheless, oligodendrocyte precursors are unique among glial cells in that they receive direct synaptic input from different types of neurons; whether OPC also send signals to neurons is still unknown. Here we show a bidirectional communication between neurons and oligodendrocyte precursors: neuronal activity regulates the cleavage of a glial membrane protein and the release of an extracellular domain that in turn modulates synaptic transmission between neurons. Our data thus show that a particular subtype of glial cells, oligodendrocyte precursors, functionally integrate into the neuronal network and we link this bidirectional signaling to mouse behavior and disease.

## Introduction

Oligodendrocyte precursor cells (OPC) in the mammalian central nervous system (CNS) characteristically express the chondroitin sulfate proteoglycan nerve-glia antigen 2 (NG2) (SwissProt Q8VHY0), a type-1 membrane protein [1]–[5]. In contrast, NG2 expression is lacking in other glia and neurons. These NG2+ OPC represent 5%–8% of total cells in the adult brain [6],[7] and are ubiquitously spread throughout the grey and white matter: they are unique among glia in forming glutamatergic and GABAergic synapses with neurons [8],[9]. These neuron-OPC synapses are present in all major brain areas including hippocampus, cerebellum, corpus callosum, and cortex [10]–[14]. Differentiation of OPC into oligodendrocytes is associated with a down-regulation of NG2 expression and a loss of synapses in spite of the retention of functional glutamate (Glut) receptors [15],[16]. OPC respond to neuronal activity; recent studies showed that OPC differentiation and migration [17],[18], as well as myelination appear to be under the control of neuronal activity [19]–[22].

Definition of the underlying molecular mechanisms by which neuronal activity influences OPC (reviewed in [23],[24]), as well as feedback mechanisms enabling OPC to respond to and potentially modulate neuronal activity, has remained elusive (reviewed in [25]). Studies to date have only described a unidirectional communication between neurons and OPC at synapses [8],[26]. The NG2 protein contains two neurexin-like (lamininG-neurexin-sex hormone binding globulin [LNS]) domains at the N-terminus [27], suggesting it may function at synapses similar to LNS domain containing neurexins [28],[29]. Moreover, the intracellular C-terminus has a PDZ-binding motif, which binds the intracellular α-amino-3-hydroxy-5-methyl-4-isoxazolepr opionicacid (AMPA) receptor-binding PDZ protein GRIP and may orientate OPC AMPA receptors (AMPARs) at sites of neuronal contact [30].

An NG2 ectodomain has been reported to be extractable in aqueous buffers from the extracellular matrix (ECM), suggesting that NG2 cleavage occurs *in vivo* in the normal CNS [31],[32]. Recent results demonstrated activity-dependent cleavage of the synaptic neuronal adhesion molecules N-cadherin and neuroligin1 by the α-secretase ADAM10 with subsequent modifications of synaptic structure and function [33],[34]. Additionally, postnatal deletion of neuronal ADAM10 activity resulted in epileptic seizures, learning deficits, altered spine morphology, and synaptic dysfunction [35]. Cleavage by α- and γ-secretases and signaling properties of the fragments generated has been best characterized for the Notch protein, where a membrane bound C-terminal fragment (CTF) and an intracellular domain (ICD) are generated [36],[37].

In the present study, we show that NG2 is constitutively cleaved by *α*- and γ-secretases, leading to a 290 kD ectodomain as a major product, a membrane-associated CTF of 12 kD, and a short ICD of 8.5 kD. *In vivo* cleavage and release of the NG2 ectodomain into the ECM can be increased by distinct stimuli increasing network activity. In many cases these stimuli directly promote NG2 cleavage in isolated primary OPC. Inhibition of ADAM10 blocks both this activity-dependent release and also the constitutive cleavage of the ectodomain, identifying ADAM10 as the major protease cleaving NG2 under physiological conditions. To elucidate the functional role of NG2 cleavage and the OPC-derived NG2 ectodomain, we studied the electrophysiology of pyramidal neurons in the somatosensory cortex in mice lacking the NG2 protein (NG2^−/−^ mice, NG2-knockout). We observed a strong impairment of N-methyl-D-aspartate receptor (NMDAR)-dependent long-term potentiation (LTP) and reduced Glut receptor currents at these neuronal synapses. Furthermore, we observed that inhibition of ADAM10 in control mice mimics the NG2^−/−^ phenotype, reflected by a reduction in NMDAR-dependent LTP. Neuronal AMPAR- and NMDAR-mediated currents are reduced in NG2^−/−^ mice, while AMPAR but not NMDAR reveal an altered subunit composition at neuronal postsynapses in NG2^−/−^ mice: strikingly altered AMPAR characteristics could be restored to the wild-type (WT) pattern by addition of recombinant protein comprising the conserved N-terminal LNS domains of the NG2 ectodomain to the slices. These NG2 domains also increased levels of c-fos in Glut-stimulated slices of the somatosensory cortex, demonstrating that they affect activity-dependent gene expression in target neurons. NG2^−/−^ mice reveal altered behavior in tests measuring sensorimotor function.

Taken together, our data demonstrate for the first time that OPC not only respond to, but are also able to modulate the neuronal network. They show that the NG2 protein itself is part of this regulatory mechanism, via activity-dependent cleavage and release of the NG2 ectodomain from OPC. Furthermore, we identify the two conserved LNS domains of the NG2 ectodomain as potent modulators of neuronal activity and gene expression.

## Results

### ADAM10 and γ-Secretase Dependent Cleavage of NG2

**Figure 1:**
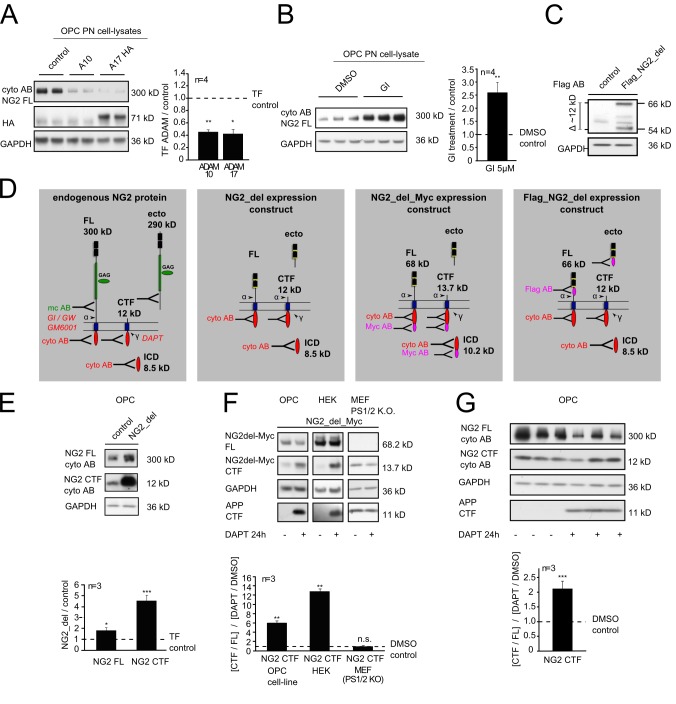
NG2 is cleaved by α- and γ-secretases. (A, B) Immunoblot analysis of endogenous NG2 FL protein of an OPC cell line using an antibody against the ICD of NG2 (cyto AB) (see Figure S1). (A) Reduction of NG2 FL protein levels after overexpression of ADAM10 or 17. (B) NG2 FL levels were increased if endogenous ADAM10 was inhibited by GI. (C) Immunoblot analysis of the Flag_NG2_del construct after expression in an OPC cell line using an antibody against the Flag tag within the ectodomain. Major protein bands were detected at 66 kD (FL protein) and 54 kD (largest ectodomain). (D) Schematic illustration of the murine NG2 FL protein and NG2_del expression constructs used. Cleavage sites, fragments, and epitopes recognized by antibodies (AB) are indicated. (E) Immunoblot analysis of endogenous compared to the NG2_del derived CTF. NG2 CTF levels are increased after expression of the NG2_del construct in an OPC cell line, both CTF run at the same molecular weight of 12 kD. (F) Immunoblot analysis of NG2_del_Myc expression in three different cell lines. Application of the γ-secretase inhibitor DAPT highly increased Myc-tagged CTF in OPC and HEK cells. In MEF cells that are lacking presenilin 1 and 2, DAPT application was without effect. CTF levels of APP were used as a control to show the inhibition of γ-secretase activity by DAPT. (G) Immunoblot analysis of endogenous NG2 CTF levels in an OPC cell line after inhibition of the γ-secretase with DAPT. CTF levels were increased when DAPT was present and normalized against the FL protein levels. (In (A), *n* represents four experiments each performed in duplicate, the WB shows one experiment. In (B) and (G) each experiment consists of triplicates. Data represent the mean ± SEM (paired Student's *t* test).

To analyze NG2 processing, we used a well-characterized cell line of murine OPC (Oli-neu) [38]. These cells, in contrast to primary OPC (pOPC), can be readily transfected and represent a good model system, since all cells express endogenous NG2 and are arrested in their differentiation. Overexpression of the α-secretases ADAM10 (A10) and ADAM17 (A17, TACE) in OPC reduced by over 2-fold the levels of full-length (FL) NG2 (300 kD, cyto AB) (see Figure 1D) in post nuclear (PN) cell-lysates after 48 h (Figure 1A). Culture of OPC for 6 h with the specific ADAM10 inhibitor GI254023X (GI) resulted in a 2.5-fold increase in levels of the FL protein (Figure 1B).

To visualize size differences due to α-secretase-dependent shedding of NG2 more easily, a shortened recombinant form of NG2, NG2_del with a Flag tag in the extracellular domain was expressed (Flag_NG2_del) (Figure 1C) [39]. Lysates analyzed for the largest protein fragments, revealed the FL (66 kD) and the α-secretase cleavage product, an ectodomain of 54 kD (Figure 1C), analogous to the 300 kD FL and 290 kD ectodomain of endogenous NG2.

Next we investigated the generation of a membrane bound CTF of NG2 and its cleavage by the γ-secretase complex. In OPC expressing NG2_del (Figure 1D), levels of the corresponding CTF fragment increased 4 times compared to endogenous CTF in control cells with no NG2_del expression (Figure 1E). Additionally the endogenous NG2 CTF runs at exactly the same size as the CTF from NG2_del (∼12 kD), again verifying an α-secretase-mediated cleavage of NG2 and NG2_del at the same site and the creation of an 290 (or 54) kD ectodomain.

To distinguish overexpressed from endogenous NG2 CTF, expression of NG2_del with an intracellular c-terminal Myc tag (Figure 1D) was analyzed after expression in three different cell lines. γ-secretase activity was inhibited by application of DAPT for 24 h, resulting in a further increase of Myc tagged NG2 CTF (Figure 1F). In OPC, levels of the Myc tagged NG2 CTF increased 6-fold when the γ-secretase was inhibited. In human embryonic kidney 293 (HEK) cells the CTF levels increased 12-fold in the presence of DAPT. Incubation of the γ-secretase inhibitor with a mouse embryo fibroblast (MEF) line, which lacks γ-secretase activity owing to a genetic deletion of the presenilin 1 and 2 genes, did not affect levels of the NG2 CTF. Increased CTF levels of amyloid precursor protein (APP) were used as a control to demonstrate the inhibition of γ-secretase activity by DAPT (Figure 1F and 1G).

These results demonstrate γ-secretase-mediated cleavage of the NG2_del derived CTF. Similarly, culture of untransfected OPC for 24 h in the presence of DAPT resulted in a 2-fold increase of CTF levels derived from the endogenous NG2 (Figure 1G). As reported originally by the Barres group [40], we also observed that culture in the presence of DAPT caused an accelerated differentiation of OPC as indicated by a down-regulation of NG2 protein expression (Figure 1G).

### Sequential Cleavage Generates the NG2 CTF and ICD

**Figure 2:**
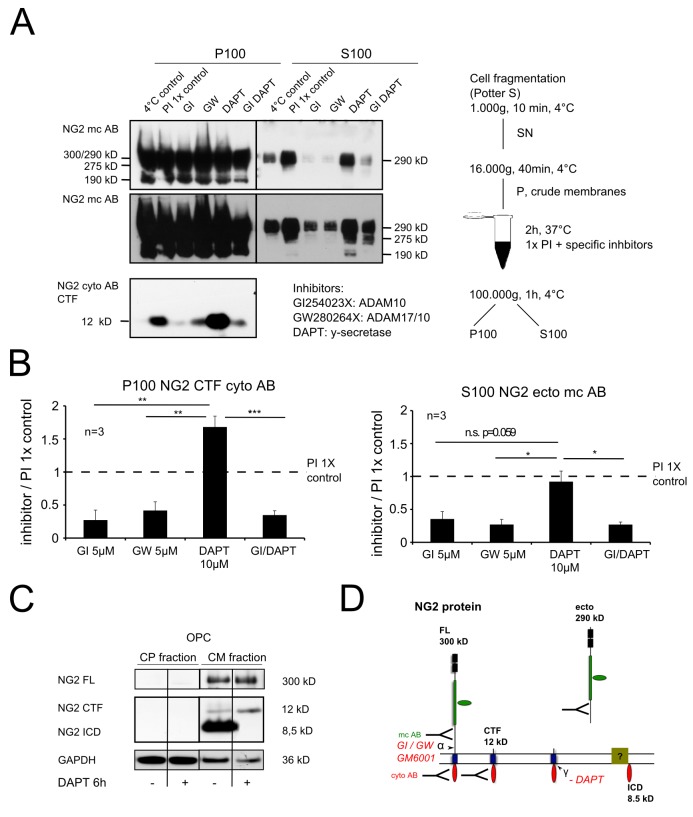
NG2 ectodomain cleavage is obligatory for generation of the CTF and ICD. (A) Immunoblot analysis of NG2 CTF (P100, membrane fraction) and ectodomain (S100, soluble fraction) levels. The lower picture represents a longer exposure time of the same blot. (B) Quantification shows a decrease of CTF levels when α-secretases ADAM10 and 17 are inhibited (GI or GW), but an increase if only the γ-secretase is inhibited (DAPT). Inhibition of α- and γ-secretase (GI DAPT) had the same effect as α-secretase inhibition alone. Levels of the soluble ectodomain are lower in the S100 fraction when α- or α- and γ-secretase are inhibited (GI, GW, or GI DAPT) but are not altered if only the γ-secretase is inhibited (DAPT) (paired Student's *t* test). (C) Endogenous NG2 ICD was detectable in a crude membrane fraction in the absence of DAPT. (D) Schematic illustration of sequential NG2 cleavage, fragment localization, and antibodies used.

To further analyze the sequential cleavage of NG2 by *α*- and γ-secretases we used a cell-free assay. Purified crude membranes (CMs) from OPC were incubated for 2 h with different combinations of specific protease inhibitors (GI, ADAM10 inhibitor; GW, A17/10 inhibitor and DAPT, γ-secretase inhibitor) and compared to the control with standard protein inhibitors (PIs; 1× control). Membrane associated proteins (P100) were subsequently separated from soluble proteins (S100) generated within the 2 h of incubation by ultracentrifugation (Figure 2A). A protein band at 300/290 kD was detectable in P100 representing full length NG2 protein and the 290 kD ectodomain (mc AB) (see Figure 2D). Additional major bands of truncated but high molecular proteins were detectable at 275 and 190 kD, which are likely to be additional ectodomains. In S100 all three theoretical ectodomain fragments were detected, although the levels were lower compared to forms of similar size in P100, suggesting that substantial amounts of the NG2 ectodomain bind to the membrane. The ectodomain origin of these cleavage forms was supported by their failure to react with the NG2 cyto AB (Figure S6A). Application of α-secretase inhibitors (GI or GW) alone or in combination with the γ-secretase inhibitor (GI and DAPT), led to a significant reduction of the 290 kD ectodomain in S100 (Figure 2B). Incubation with DAPT alone had no effect. The generation of the NG2 CTF (present in the P100 fraction only) (Figure 2A) was also influenced by the inhibitors: GI or GW as well as GI and DAPT reduced CTF levels while DAPT alone increased the CTF levels (Figure 2B). These results demonstrate that the α-secretase ADAM10 is responsible for the majority of the shedding in this paradigm, and that the ectodomain shedding is independent of γ-secretase activity. Furthermore, α-secretase cleavage is mandatory for subsequent cleavage by the γ-secretase. Incubation of the membranes with inhibitors of the β-secretase had no effect, excluding the activity of the β-secretase in this process (Figure S6B). The NG2 ICD was enriched in a crude-membrane fraction from Oli-neu (Figure 2C) and levels were decreased by γ–secretase inhibition, suggesting a role in a membrane-associated complex (Figure 2C and 2D).

### Activity-Dependent Proteolytic Cleavage of NG2 Results in Increased Levels of Ectodomain Associated with the ECM

**Figure 3:**
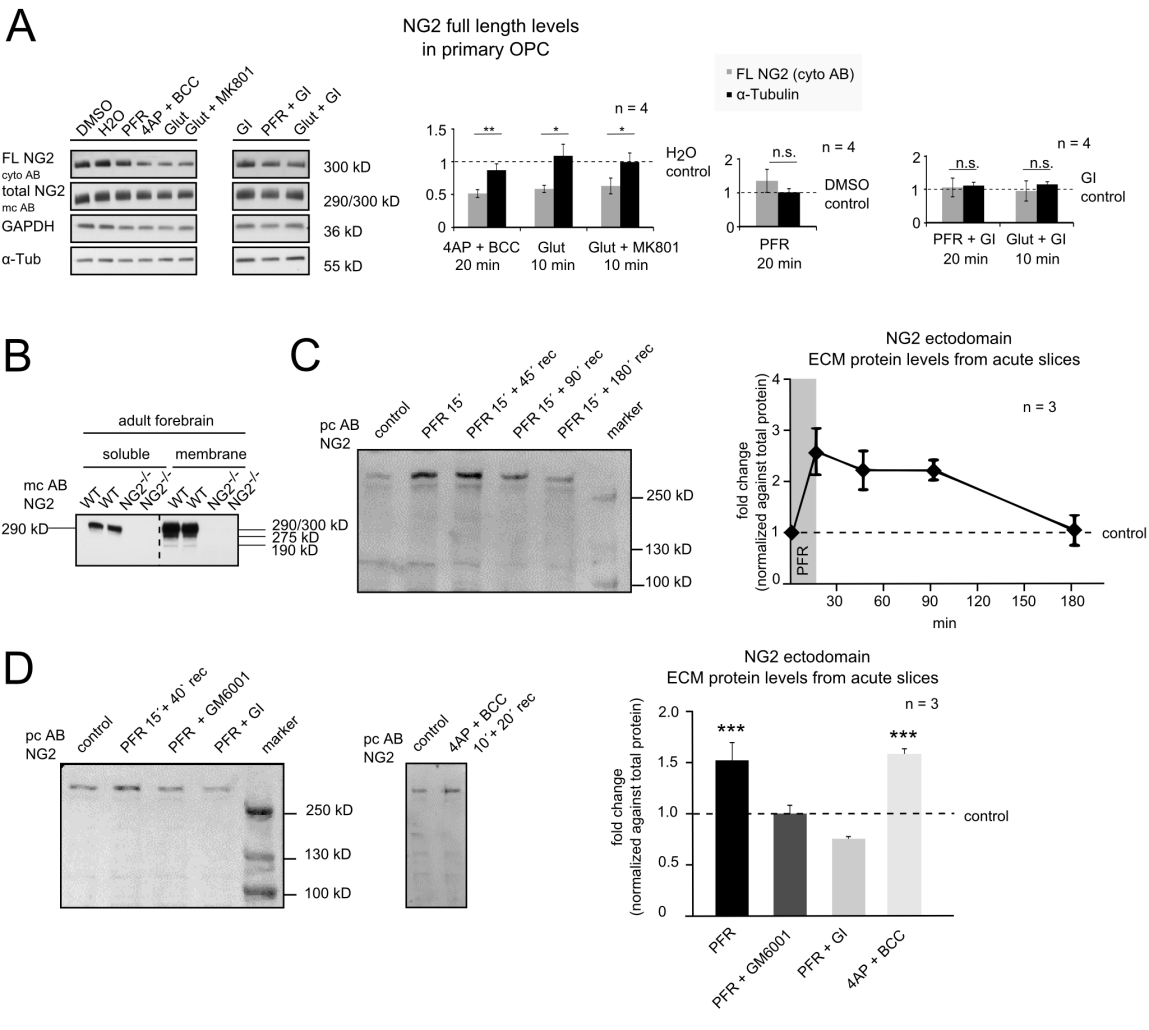
Activity-dependent NG2 cleavage and release of the ectodomain into the ECM. (A) Primary OPC were isolated from total mouse brain of postnatal day 9 (P9) by magnetic cell sorting (MACS). After 1 day of culture (90%–95% OPC), the cells were incubated with PFR or 4AP + BCC for 20 min or with Glut for 10 min. To test the involvement of NMDARs, in some experiments cells were pre-incubated for 15 min with the NMDAR inhibitor MK801 (Glut+MK801) and to confirm shedding by ADAM10 with its inhibitor GI (PFR + GI, Glut + GI). Cells were harvested and PN cell-lysates were obtained by differential centrifugation prior to subjecting to Western blot. Western blot quantification (bar graphs) revealed a reduction in the NG2 FL protein for 4AP + BCC, Glut, and Glut+MK801 stimulation, no change was observed if the cells were stimulated with PFR or if the cells were pre-incubated with the ADAM10 inhibitor GI (unpaired Student's *t* test). (B) Soluble (saline) and membrane fractions (triton) were obtained from forebrains of adult WT and NG2^−/−^ mice. Only a small amount of the NG2 290 kD ectodomain is soluble in the saline-fraction in the WT (see Figure S1) as reported before. (C) Western blot of ECM extracts of acute hippocampal slices from P70 rats by digestion of the glycosaminoglycan chains by chondroitinase ABC without detergent. An increase of the NG2 ectodomain was detected after chemical LTP (PFR) stimulation for 15 min (15′). The NG2 ectodomain levels within the ECM return to starting levels after 180 min of recovery time. NG2 ectodomain levels were normalized against total protein from a matching coomassie gel (Figure S5). (D) NG2 ectodomain levels increase 1.5-fold 15 min after stimulation with PFR and 40 min recovery time detected by Western blot from ECM extracts of acute hippocampal slices. Inhibition of metalloproteases (GM6001), or ADAM10 (GI), prevented the increase of ECM-associated NG2 ectodomain. Incubation with GI reduced levels of ECM-associated NG2 ectodomain to below the control value. Treatment of the slices with 4AP + BCC for 10 min with 20 min recovery time showed the same increase in levels of ECM-associated NG2 ectodomain as seen with the PFR treatment (one way ANNOVA with Dunnett's multiple comparison test).

It has recently been reported that cleavage of the synaptic cell adhesion molecule neuroligin1, expressed by neurons, is under the control of electrical activity with modulatory effects on synaptic function [34],[41]. To investigate if NG2 cleavage is similarly regulated, primary OPC from P9 mouse brain were isolated by magnetic beads coupled to monoclonal antibody recognizing NG2. After 1 day of culture, the cells were incubated with (I) picrotoxin, forskolin, and rolipram (PFR) inducing chemical LTP (cLTP) for 20 min; (II) 4-aminopyridine and bicuculline (4AP + BCC), a weak potassium and a GABA_A_ channel blocker, known to depolarize neurons, for 20 min; (III) Glut for 10 min. In some cases the cells were pre-incubated with the NMDAR antagonist MK801 prior to incubation with Glut, or with the ADAM10 inhibitor GI for 15 min prior to stimulation with PFR or Glut. We observed a significantly reduced level of NG2 FL protein in PN cell lysates after incubation with 4AP + BCC, and after incubation with Glut or Glut+MK801 (Figure 3A). PFR treatment did not alter NG2 FL levels. After pre-incubation with the ADAM10 inhibitor GI, neither Glut nor PFR altered NG2 FL levels. These results imply that ADAM10 cleavage-dependent reduction of NG2 FL protein can be increased by activity directly in primary OPC.

The NG2 ectodomain of 290 kD can be extracted with PBS from the ECM of the rodent brain (Figures 3B and S1) [31],[32]. To analyze the effect of activity-dependent effects of the whole neural network on NG2 cleavage within OPC, we subjected acute hippocampal slices to cLTP (PFR incubation) or incubation with 4AP + BCC and analyzed levels of the released NG2 ectodomain. Here we extracted ECM proteins by 30 min incubation with chondroitinase ABC, which removes exclusively sugar side chains from proteins, subsequent to the stimulation. The resulting samples contain only soluble, extracellular proteins as membrane proteins are not extracted in the absence of detergent, similar to the extraction of the ectodomain from tissue with PBS described above but with less mechanical stress. These samples thus contain only shedded soluble NG2 ectodomain. Strikingly, levels of the NG2 ectodomain were increased 2.5-fold after 15 min of PFR (Figure 3C). Levels slightly decreased with ongoing recovery time and returned to starting levels after 180 min of recovery time (Figure 3C). In order to identify the protease responsible for the activity-dependent ectodomain shedding, we incubated the slices in the presence of the metalloprotease inhibitor GM6001 or the ADAM10 inhibitor GI. Both compounds completely inhibited the NG2 ectodomain shedding induced by PFR (Figure 3D). In the presence of the ADAM10 inhibitor GI, the ECM-associated levels of the NG2 ectodomain were reduced to below those of the control, demonstrating that basal levels of NG2 cleavage in OPC are ADAM10-dependent. Incubation of acute slices with 4AP + BCC, which depolarizes neurons and affects OPC as described above, increased NG2 ectodomain levels in the ECM in a comparable fashion to incubation with PFR (Figure 3D).

### Mice Lacking NG2 in OPC Exhibit Impaired NMDAR-Dependent LTP at Neuronal Synapses

**Figure 4:**
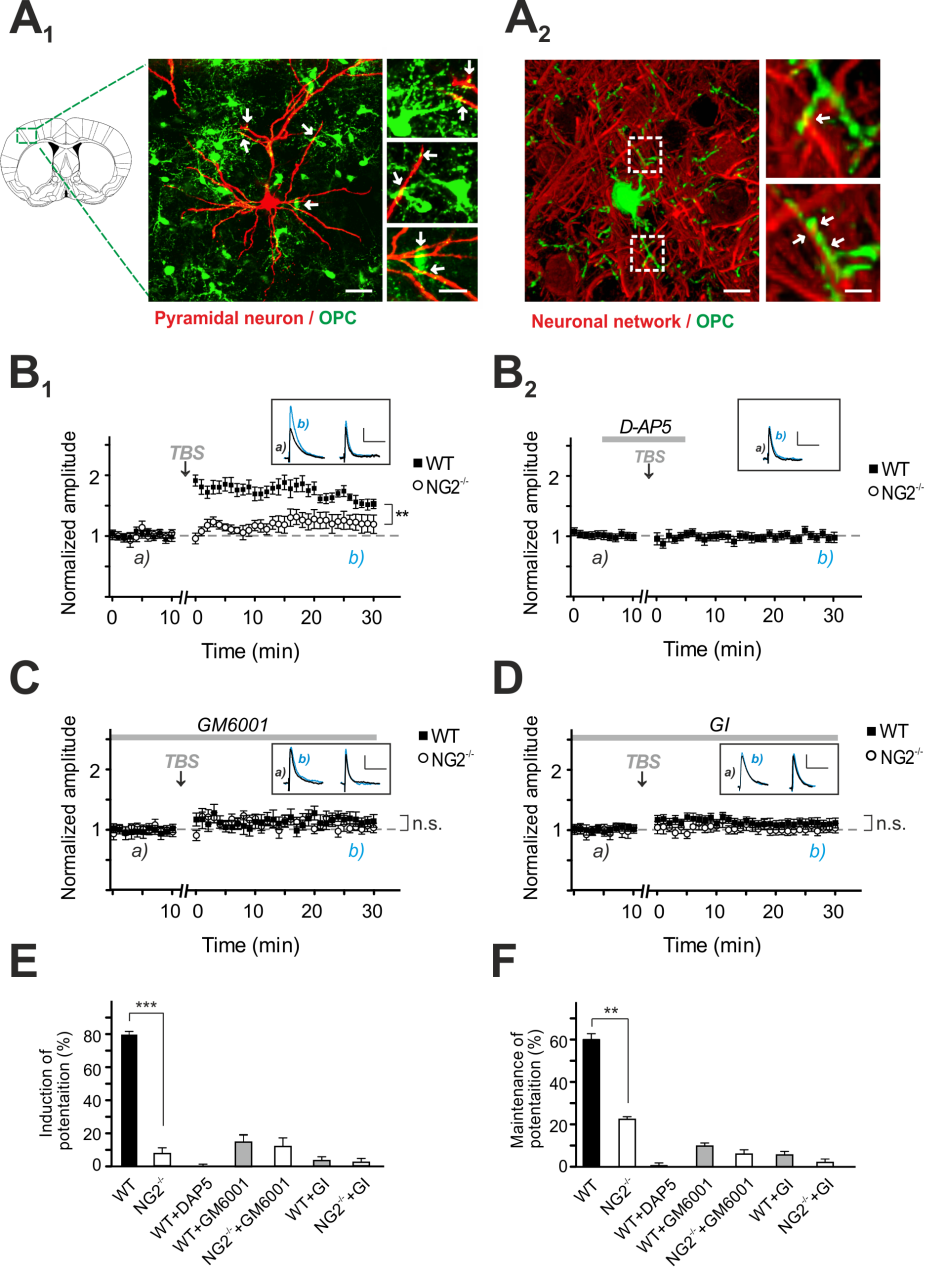
NMDAR-dependent LTP is impaired in NG2^−/−^ mice and after inhibiting proteases in control slices. (A) A_1_: Image of L2/3 pyramidal cell filled with biocytin (red) contacted by OPC expressing EYFP (green: NG2^−/−^-EYFP^+/+^ cells) as indicated by white arrows. Scale bars represent 25 µm and 10 µm in higher magnifications. A_2_: Image of a cortical OPC (green: NG2^−/−^-EYFP^+/+^ cells) and the surrounding neuronal network (red: SMI31, MAP-2, NeuN). Intimate contacts between both structures are highlighted. The image represents a 3D volume render of a confocal stack. Scale bars represent 10 µm and 2 µm in higher magnifications. (B_1_–B_2_) NMDAR-dependent LTP at L4-to-L2/3 synapses of pyramidal neurons in the somatosensory cortex of control mice (filled symbols) and NG2^−/−^ mice (open symbols). The timepoint of LTP inducing TBS is indicated by black arrows. Small inserts show EPSPs at the indicated times (a and b). A block of NMDARs by D-AP5 application is indicated by the grey line (B_2_) (paired Student's *t* test). (C) NMDAR-dependent LTP in the presence of the metalloprotease inhibitor GM6001 (paired Student's *t* test). (D) NMDAR-dependent LTP in the presence of ADAM10 inhibitor GI (paired Student's *t* test). (E) Summary graph of NMDAR-dependent EPSPs after 1–5 min post-TBS (unpaired Student's *t* test). (F) Summary graph of the relative potentiation of NMDAR-dependent EPSPs at 20–30 min after TBS (unpaired Student's *t test*).

Next we investigated a potential effect of the cleaved NG2 ectodomain on neuronal synaptic transmission. To address this question, we used transgenic mice with ablation of NG2 (NG2^−/−^). In these mice OPC do not express NG2 (NG2^−/−^ OPC) [42]. Whole-cell recordings were made from L2/3 pyramidal neurons in the somatosensory cortex; these are contacted by NG2^−/−^-OPC that express enhanced yellow fluorescent protein (EYFP) (green in the Figure 4A). We analyzed NMDAR-dependent LTP as a typical form of synaptic long-term plasticity.

Cortical pyramidal neurons were current-clamped at a membrane-potential of −80 mV, and excitatory postsynaptic potentials (EPSPs) were evoked by electrical stimulation in layer IV that stimulated ascending afferent fibers projecting onto these pyramidal neurons. Baseline EPSPs were initially recorded for 10 min before applying an LTP inducing stimulation protocol that consisted of presynaptic high-frequency theta-burst stimulus (TBS) paired with postsynaptic depolarization. This protocol is known to induce high presynaptic glutamate release as well as an enhanced opening of postsynaptic glutamate receptors due to the membrane depolarization [43].

This LTP stimulation protocol, induced an increase of the EPSP amplitude of EPSPs in pyramidal neurons of WT control mice up to 79.4%±2.8% within 5 min post-TBS (Figure 4B_1_ and 4E); these were maintained for up to 60.1%±6.2% after 20–30 min post LTP induction (TBS) (Figure 4B_1_ and 4F). To test the strength of neuronal synaptic plasticity associated with the absence of NG2, we repeated the LTP experiment at the same synaptic connections in NG2^−/−^ mice. Strikingly, in neurons of NG2^−/−^ mice only a minor potentiation of EPSPs was detected within 5 min post-TBS (7.2±2.6) (Figure 4B_1_ and 4E), which was maintained up to 23%±2.9% after 20–30 min post-TBS (Figure 4B_1_ and 4F). The NMDAR-dependency of this form of LTP was confirmed by bath application of D-AP5 (which blocks NMDAR) during TBS in controls, resulted in no potentiation (0.19%±2.51%) (Figure 4B_2_).

### Inhibition of ADAM10 Activity Results in a Similar Impairment of NMDAR-Dependent LTP at Neuronal Synapses to That Seen in NG2^−/−^ Mice

ADAM10 is the major metalloprotease responsible for NG2 cleavage, whose activity is promoted by stimulation of the neural network. To examine the physiological relevance of NG2 ectodomain processing by ADAM10 for neuronal activity, we used the metalloprotease blocker GM6001 and the ADAM10-specific inhibitor GI, which we had shown prevents activity-dependent release of the NG2 ectodomain (Figure 3A and 3D) and repeated LTP recordings in cortical slices from control (WT) and NG2^−/−^ mice. Following a pre-incubation period of 1 h, GM6001 abolished the induction of NMDAR-dependent LTP in controls and mimicked a NG2^−/−^-like phenotype, as reflected by a small EPSP increase of 9.8%±3.3% after 20–30 min post LTP induction by TBS (Figure 4C). GM6001 did not alter the level of EPSPs in neurons of NG2^−/−^ mice (20–30 min post-TBS: 6%±2.3%) (Figure 4C). Similar results were obtained when the experiments were repeated substituting GI for GM6001, which also prevented LTP induction in neurons of control mice (5.6%±1.3%), but did not affect neurons of NG2^−/−^ mice (2.5%±2.0%) (Figure 4D). These data suggest that the ectodomain of NG2 is released by the action of the metalloprotease ADAM10 in the vicinity of neuronal synapses and can modulate glutamatergic transmission between cortical pyramidal neurons. Subsequently we examined intrinsic membrane properties of L2/3 pyramidal neurons and found no differences in membrane capacitance and input resistance after NG2 deletion (Figure S2), thereby most likely excluding influences of altered ion channel conductance on the cellular excitability in NG2^−/−^ mice. Interestingly pyramidal neurons of NG2^−/−^ mice displayed a slightly hyperpolarized resting membrane potential (ΔV_m_ = −3.3±0.1 mV) (Figure S2).

### Decreased Neuronal Excitability Results from Altered Composition of Neuronal Glutamate Receptors

We examined basal glutamatergic neurotransmission at L4-to-L2/3 synapses in NG2^−/−^ mice and recorded spontaneous excitatory postsynaptic currents (sEPSCs). These signals were exclusively mediated by glutamatergic AMPA receptors, since we blocked the activation of GABA- and NMDARs by bath application of PTX (50 µM) and D-AP5 (25 µM), respectively. We found no differences in NG2^−/−^ mice compared to WT, the mean frequency and peak amplitude of sEPSCs were not altered in L2/3 pyramidal neurons (Figure S3). Next we assessed paired pulse facilitation of synaptic currents to detect potential changes of presynaptic glutamate release. The paired pulse ratio, which was calculated from two consecutive evoked EPSCs, was similar in NG2^−/−^ and control mice (Figure S4). Thus, together with unchanged sEPSCs, these data suggest that presynaptic glutamate release is unaffected in neurons of NG2^−/−^ mice.

**Figure 5:**
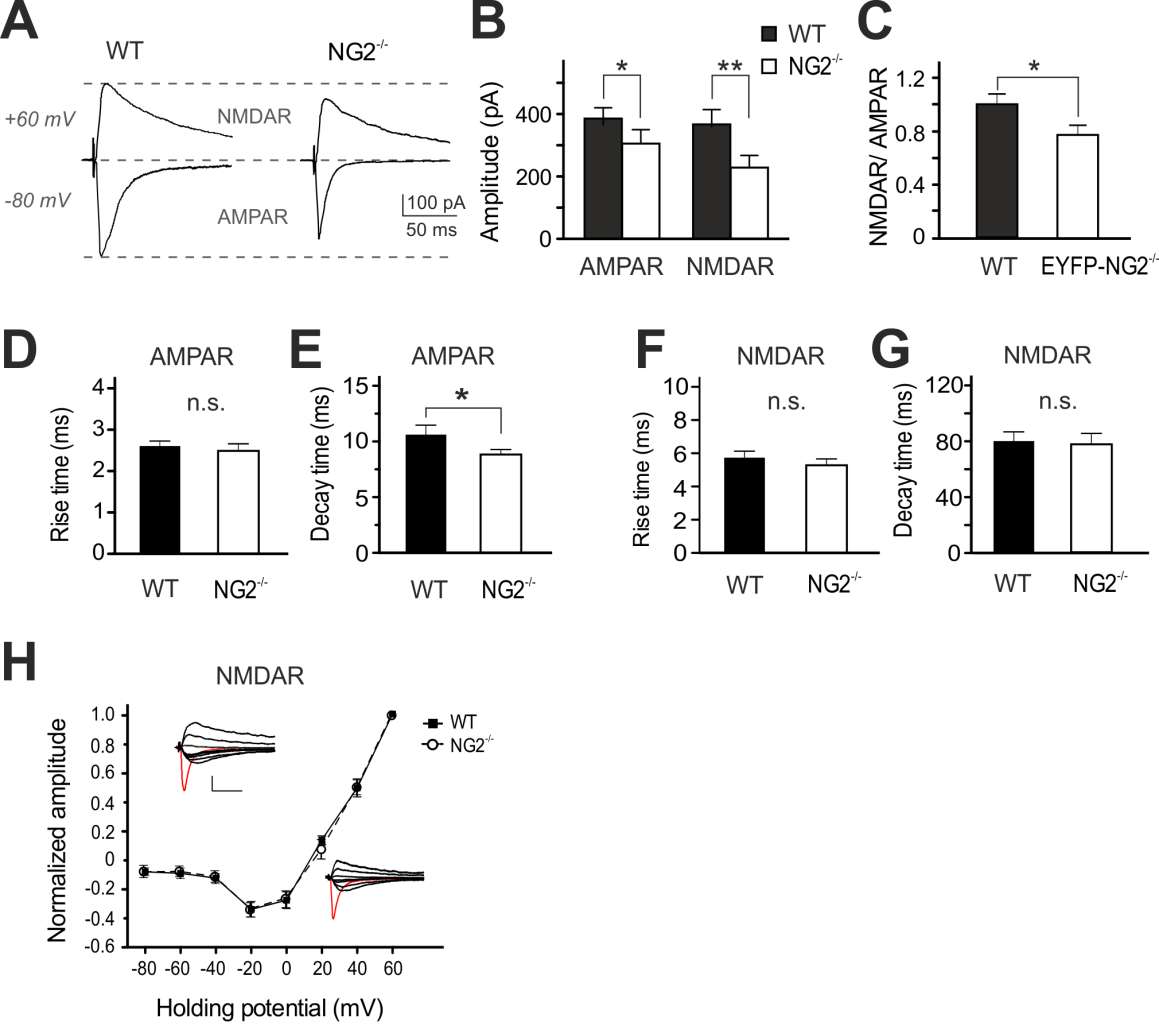
Reduced glutamatergic receptor mediated EPSCs at neuronal synapses of NG2^−/−^ mice. (A) Example traces show AMPAR-EPSCs and NMDAR-EPSCs detected at a holding potential of −80 and +60 mV, respectively. (B) Summary plot showing average AMPAR- and NMDAR-EPSC amplitudes (unpaired Student's *t* test). (C) Average ratio of pharmacologically isolated NMDAR- to AMPAR-currents, recorded at holding potential of +60 mV and −80 mV, respectively (unpaired Student's *t* test). (D–G) Summary plots of rise times (D, F) and decay time constants (E, G) of AMPAR and NMDAR currents (unpaired Student's *t* test). (H) Current-voltage (*I-V*) relations of NMDAR currents at L4-to-L2/3 synapses (unpaired Student's *t* test). In all cases, WT and NG2^−/−^ neurons are compared.

NMDAR-dependent LTP is often associated with a reduction in the number or changes in the subunit composition of postsynaptic glutamate receptors [42],[43]. To examine the functional properties of glutamatergic AMPARs and NMDARs at excitatory synapses, we recorded AMPAR- and NMDAR-currents within the same neuron. AMPAR-mediated currents were recorded at −80 mV, a potential where NMDARs are rarely open, followed by detection of NMDAR currents at +60 mV in the presence of DNQX (20 µM), which blocks AMPAR activity. Strikingly, both types of receptor currents were reduced in their amplitudes in NG2^−/−^ mice, with AMPAR currents being impaired by 24.5%±2% compared to controls and the NMDAR current component being decreased by 43.7%±2.2% (Figure 5A and 5B). Accordingly, we observed a shift in the ratio of NMDARs to AMPARs (NG2^−/−^: 0.8±0.07 versus control: 1.0±0.1) (Figure 5C). However, despite the drop in the amplitude of NMDAR currents, the kinetic properties (Figure 5F and 5G) and the current-voltage relation of NMDAR currents (Figure 5H) remained unchanged with NG2 deletion, suggesting no alteration of subunit composition. In contrast, one kinetic parameter of AMPAR currents (Figure 5D and 5E), the decay time constant, was significantly decreased in neurons of NG2^−/−^ mice (8.9±0.4 ms) compared to controls (10.7±0.7) (Figure 5E), indicating an altered subunit composition of AMPARs but not NMDARs in the NG2^−/−^ mice.

### Postsynaptic Currents at L4-to-L2/3 synapses of Pyramidal Neurons Reveal a Change of AMPAR Subunit Composition in NG2^−/−^ Mice

**Figure 6:**
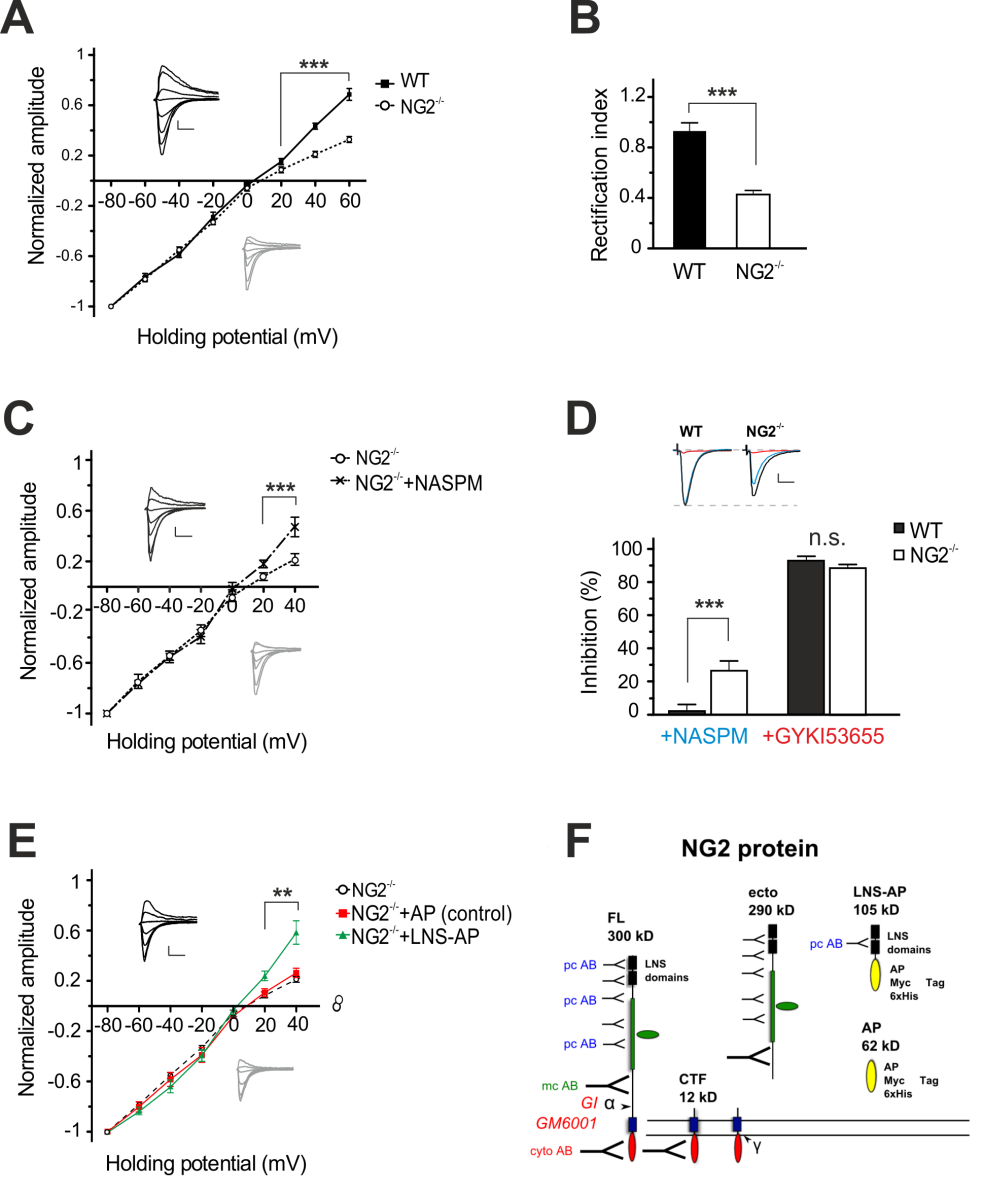
Synaptic currents in cortical pyramidal neurons of NG2^−/−^ mice reveal properties of Ca^2+^-permeable AMPARs. (A) Current-voltage (*I-V*) relations and respective current responses of AMPARs in control (filled symbol/black lines) and NG2^−/−^ cells (open circle/grey lines) (unpaired Student's *t test*). (B) Rectification index of AMPAR currents at +40 and −80 mV (unpaired Student's *t* test). (C) Current-voltage (*I-V*) relations of AMPAR currents in pyramidal cells of NG2^−/−^ mice before (open circle/black line) and after (cross symbol/dashed line) inhibition of Ca^2+^-permeable AMPARs with NASPM (paired Student's *t* test). (D) Summary plot showing percentage of inhibition of AMPAR currents blocked either by NASPM (blue) and GYKI53655 (red) and respective example traces (top panel) (unpaired Student's *t* test). (E) Current-voltage (*I-V*) relation of AMPAR currents in pyramidal cells of NG2^−/−^ mice (open circle/black broken line) pre-incubated for 1 h with the control AP protein (square/red line) or the NG2 LNS-AP protein (triangle/blue line). Only LNS-AP protein treated slices influenced the *I-V* relationship resulting in a linear *I-V*-curve at positive membrane potentials similar to the one exhibited by neurons of WT-mice, shown in (A) (unpaired Student's *t* test). (F) Schematic illustration of the complete NG2 protein and the LNS-AP (105 kD) and AP (62 kD) recombinant proteins. LNS-AP consists of the two N-terminal LNS domains of the NG2 ectodomain, with a C-terminal AP, a Myc, and a hexa-His tag. A Myc hexa-His tagged AP was used as control.

To further investigate the functional properties of AMPARs at L4-to-L2/3 synapses of pyramidal neurons, we assessed a typical current-voltage (*I-V*) relationship of AMPAR-mediated currents. Strikingly, we found a strongly decreased slope, also called inward-rectification, of the resulting *I-V* curve at positive potentials in the NG2^−/−^ mice, while WT controls displayed a nearly linear *I-V* relationship (Figure 6A). The rectification index (RI) was significantly smaller in pyramidal neurons of NG2^−/−^ mice (0.4±0.03; range: 0.2–0.8) compared to controls (1.0±0.07; range: 0.6–1.6) (Figure 6B). These results suggest the presence of Ca^2+^-permeable (CP)-AMPARs at L4-to-L2/3 synapses of NG2^−/−^ mice, which was further confirmed by repeating the recordings of AMPAR currents in the presence of NASPM (250 mM), a selective inhibitor of CP-AMPAR channels [44] at a holding potential of −80 mV. Bath application of NASPM reduced the initial peak amplitude of AMPAR currents by 26.6%±8.0% in the NG2^−/−^, while AMPAR currents in controls remained unaffected (4.8%±2.1%; *n* = 6) (Figure 6C and 6D). These data suggest that both CP and Ca^2+^-impermeable AMPARs are present in the postsynaptic membrane of L2/3 pyramidal neurons from NG2^−/−^ mice. The nearly linear *I-V* relation of AMPAR currents in the presence of NASPM further substantiates this assumption (Figure 6C and 6D). Moreover, subsequent bath application of GYKI53655 (25 µM), an antagonist of all AMPAR, blocked effectively all current responses in both NG2^−/−^ (88.8%±1.8%) and control groups (91.9%±1.7%) (Figure 6D). These data confirm that the remaining current components in the presence of NASPM were mediated by AMPARs and not by other glutamate receptors like kainate receptors.

In order to investigate a potential direct influence of the NG2 ectodomain, in particular the LNS domains, on the observed AMPAR *I-V* relation in NG2^−/−^ mice, we pre-incubated cortical slices for 1 h with purified recombinant protein comprising alkaline phosphatase (AP)-tagged LNS domains of the NG2 ectodomain (LNS-AP). Strikingly, application of the NG2 LNS domains restored the AMPAR currents in NG2^−/−^ mice to WT-levels, as reflected by the presence of a normal linear *I-V* relationship (Figure 6E and 6F). In contrast a pre-incubation with the AP tag alone (control) did not alter the *I-V* relation of the AMPAR currents.

**Figure 7:**
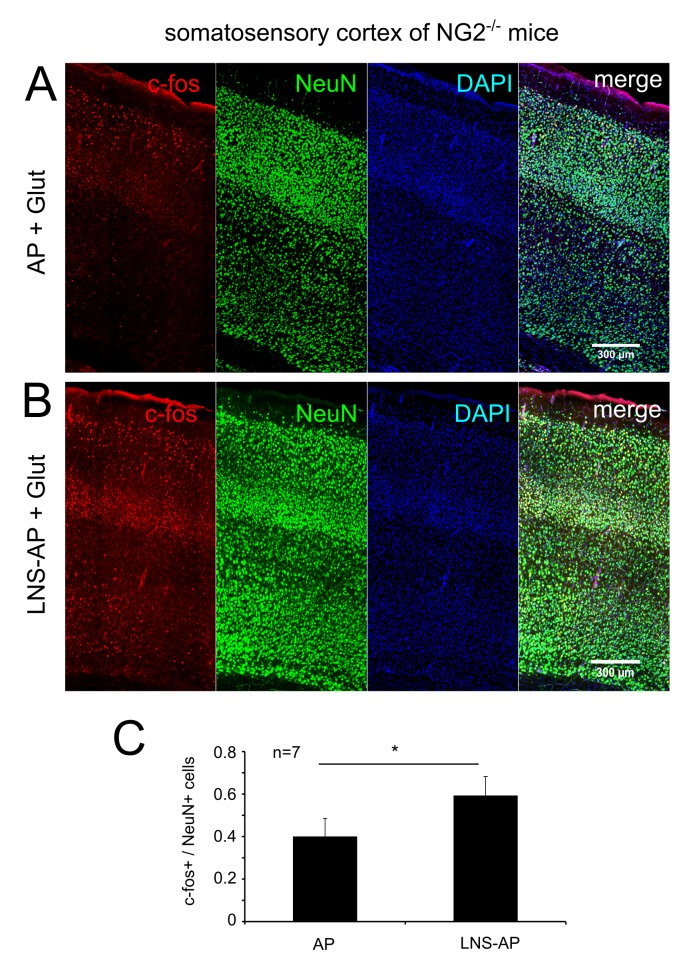
Incubation with the NG2 LNS domains results in an increased expression of c-fos in cortical neurons. (A–C) Acute slices of the somatosensory cortex of NG2^−/−^ mice were pre-incubated (1 h) with the NG2 LNS domains (LNS-AP) (B) or the AP control protein (A) and subsequently stimulated with glutamate for 10 min. Immunofluorescence staining was performed for the activity-dependent early gene c-fos (in red) and the neuronal marker NeuN+ (green). Incubation with the NG2 LNS domains resulted in more c-fos+ neurons compared to slices incubated with the control AP protein (C). c-fos+ cell numbers were related to NeuN+ cell numbers (c-fos+/NeuN+ cell numbers) from each double stained slice. Cell numbers of representative cortical areas as shown in (A) and (B) were counted from seven different animals (*n* = 7) for each incubation condition. The incubation conditions (LNS-AP and AP) were compared from the same individual mouse. c-fos/NeuN numbers were compared between the two experimental conditions (paired Student's *t* test).

Finally we also tested the effects of the NG2-LNS domains on neuronal activity in an independent approach. The expression level of c-fos, an immediate early gene and a marker for neuronal activity [45] was analyzed in cortical slices of NG2^−/−^ tissue after incubation of the slices with 1 mM Glut for 10 min to activate the neuronal network. Pre-incubation of slices with the LNS protein domains resulted in more c-fos+ neurons compared to slices preincubated with AP alone (Figure 7).

### NG2^−/−^ Mice Show Impaired Sensorimotor Gating

**Figure 8:**
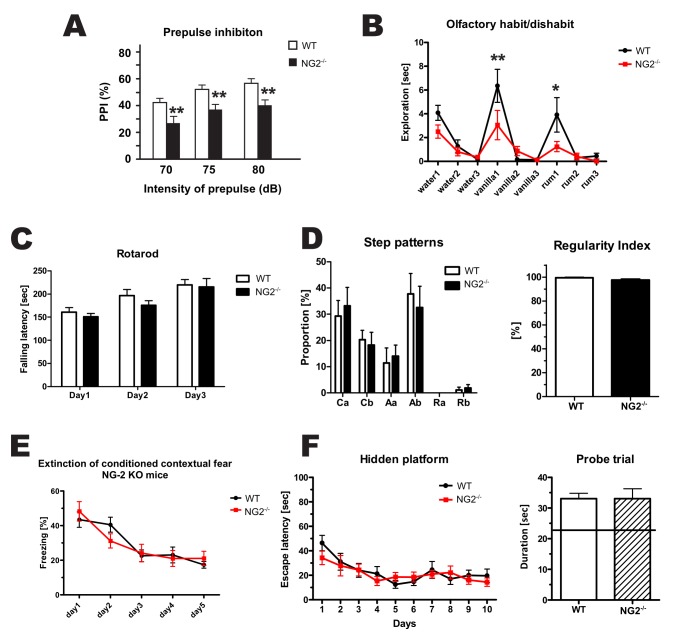
NG2^−/−^ mice show impaired sensorimotor gating. (A) PPI is reduced in NG2^−/−^ mice in comparison to WT. (B) NG2^−/−^ mice exhibited a reduced response to olfactory stimuli. Both these tests show an alteration in sensomotory gating in NG2^−/−^ mice. The Rotarod and Catwalk were applied to test the motoric of the mice. No differences were observed between the genotypes, indicating no locomotor impairment. (E) Contextual fear conditioning and (F) the Morris Water Maze test revealed no differences between the genotypes, indicating the same capacity for learning and memory. All tests were carried out with 5–8 weeks old male littermates (11 NG2^−/−^, 10 WT). (Two-way ANOVA were conducted to assess the significance of the results).

To investigate possible effects of the altered electrophysiological properties of cortical neurons in NG2^−/−^ mice on mouse behavior, we measured pre-pulse inhibition (PPI) of the acoustic startle response in these animals. In this assay, the startle response of the animals is measured after initial presentation of a weaker sensory stimulus (the pre-pulse). Normally, the response is diminished after a pre-pulse and it is thought to reveal sensorimotor gating, i.e., the ability to filter sensory input [46]. We found a significant reduction of PPI in NG2^−/−^ animals in comparison to WT littermates (Figure 8A), where the NG2^−/−^ mice still reacted strongly to the stimulus in spite of the pre-pulse. As a test for sensory input, the olfactory habituation/dishabituation test was performed. Olfactory sensations are integrated in the olfactory cortex and are a part of the somatosensory cortex (Figure 8B). The NG2^−/−^ mice spent less time sniffing the two presented odors compared to WT animals. Notably, motor cortex-associated behavior assessed by the Rotarod or CatWalk tests (Figure 8C and 8D) was unchanged in NG2^−/−^ mice. Learning and memory was tested by contextual fear conditioning (Figure 8E) and the Morris Water Maze test (Figure 8F). In both tests, no differences were observed between genotypes.

## Discussion

### α- and γ-Secretase-Mediated Cleavage of NG2

We show here that NG2 can be cleaved by α- and *γ*-secretases in a sequential manner similar to other type-1 membrane proteins such as Notch, APP, L1, N-cadherin, and neuroligin1 [33],[47]–[50]. We identified ADAM10 as the major constitutively acting α-secretase, similar to the cleavage of Notch [51]. ADAM10 cleavage of proteins generally results in two fragments, a released ectodomain and a membrane bound CTF. In the case of NG2, we found an ectodomain of ∼290 kD that is ∼10 kD smaller than the FL protein and a CTF of 12 kD. These findings match the reported NG2 ectodomain fragment of 290 kD described previously [31]. So far, CTFs or ICDs have not been reported for NG2 in contrast to the proteins described above [52],[53]. The NG2 CTF fragments were detected in membrane fractions and were more abundant when the γ-secretase was inhibited. These results suggest a functional role for the CTF fragment and demonstrate constitutive processing by the γ-secretase leading to the creation of an NG2 ICD, which was often found associated with the cell-membrane similar to the ICD of APP [54]. FL NG2 protein is expressed by OPC within the CNS and the ectodomain is likely to be contributing to the ECM [31],[55]. Interaction with collagen V&VI and β1 integrin has been shown, compatible with this concept [56],[57]. Interestingly, we found the NG2 ectodomain to be highly adhesive for OPC membranes (Figure 2A) and membrane fractions from total forebrain (Figure 3B). We also observed as reported previously, that a fraction of the 290 kD ectodomain is soluble in aqueous buffers.

Our results using isolated OPC show that NG2 is processed on OPC by endogenous ADAM10 and γ-secretase in a constitutive manner and confirm that all cleavage fragments identified originate from OPC membranes.

### Activity-Dependent Cleavage Results in Altered ECM Levels of the NG2 Ectodomain

We observed that after cleavage, part of the NG2 ectodomain is deposited in the ECM and can be extracted with chondroitinase ABC, which cleaves glycosaminogycan chains thus freeing proteoglycans from their potential association with the ECM, allowing their extraction in a soluble fraction without detergent. In our experiments ECM proteins are extracted from acute slices after incubation with activity-modulating chemicals. The amount of detergent-free extractable ectodomain rapidly increases after incubation of the slice (and thus the network), with PFR, which is known to induce cLTP by increasing cAMP levels in neurons [58]. Interestingly, ectodomain levels in this fraction returned to starting levels after 180 min, demonstrating that subsequent to shedding a fast removal of the ectodomain fragment ensues. Removal of the released ectodomain from the ECM fraction may be due to binding to cell-surface receptors and subsequent internalization or further proteolytic processing. ADAM10 appears to be the protease responsible for both the PFR-induced and constitutive cleavage *in vivo*, as ADAM10 inhibition reduced NG2 ectodomain cleavage to below control levels in the presence of PFR. Thus ADAM10 can be considered as the constitutively active protease releasing NG2 ectodomain and CTF, and neuronal activity further increases this cleavage. The activity-dependence of NG2 ectodomain cleavage can furthermore be shown by stimulation of slices with 4AP + BCC, which is reported to inhibit repolarization by blocking voltage-dependent potassium channels [59], and also increases NG2 ectodomain levels in the ECM.

Since in these treatments of acute slices we are potentially affecting all neural cell types, we also repeated the experiments with isolated primary OPC. These were subjected to the same activity-inducing chemical treatments as the slices and in addition we tested the effect of incubation with glutamate. We observed that 4AP + BCC but not the PFR treatment stimulated NG2 cleavage on isolated OPC. Incubation of OPC with the neurotransmitter glutamate additionally increased ADAM10-dependent cleavage of NG2 independent of NMDAR activity.

The combination of the effects we observed in an OPC cell line, primary OPC and the results in acute slices, suggest the following scenario. OPC have high densities of AMPAR at their synapses, which enable direct synaptic response to glutamate *in vivo* [8],[10], thus presenting a rapid signaling mechanism that potentiates ADAM10-dependent NG2 ectodomain cleavage in addition to the constitutive ADAM10-dependent cleavage. With respect to the mechanism of activity-dependent ADAM10 cleavage, we show that induction of chemical LTP (with PFR), a known stimulator of neuronal activity [58], is likely to function via the neuronal network as it did not increase NG2 ectodomain cleavage in isolated OPC. OPC exhibit voltage-dependent potassium channels [60], which could explain why 4AP + BCC incubation of primary OPC also caused an increased release of NG2 ectodomain, as blocking both voltage-dependent potassium channels and GABA_A_ receptors may lead to depolarization of the OPC, which is required for activity-dependent NG2 shedding.

In neurons, ADAM10 is located at excitatory postsynapses [61] and we have confirmed that OPC also express ADAM10. NG2 binds the PDZ domain protein GRIP in OPC, which itself binds to AMPAR forming a tripartite complex in OPC [30]. Thus an activity-dependent release of the NG2 ectodomain is likely to occur at neuron-OPC synapses resulting in increased levels of NG2 ectodomain in the ECM. These findings are similar to activity-dependent cleavage by ADAM10 of neuronal adhesion molecules such as neuroligin-1 and N-cadherin [34],[62].

### Modulation of the Neuronal Network by NG2 and ADAM10 Activity: Alterations in NMDAR-Dependent LTP

As an effect of NG2 ablation in OPC (NG2*^−/−^* OPC), we found a diminished NMDAR-dependent LTP at neuron-to-neuron synapses of pyramidal neurons in the somatosensory cortex. We observed a similar lack of neuronal LTP in neurons of control mice 45 min after blocking NG2 cleavage by ADAM10 with metalloprotease or specific ADAM10 inhibitors, while the overall small potentiation in pyramidal neurons of NG2^−/−^ mice remained unaffected by these inhibitors. These results demonstrate a major contribution of activity-dependent ADAM10 cleavage to the observed LTP changes. The neuronal cell adhesion molecule (CAM) neuroligin has recently been shown to be processed by ADAM10 in an activity-dependent manner at neuronal synapses, leading to alterations of the spine structure [34] and synaptic function. Additionally, other proteoglycans that are associated with the ECM, such as brevican [63] and tenascin R and C [64],[65], or the structural integrity of the ECM itself [66] influence long or short term potentiation at neuronal synapses. It is feasible that the released NG2 ectodomain directly interacts with a neuronal receptor, leading to modulation of glutamatergic receptor composition and glutamergic transmission at neuronal synapses. These findings are in accordance with previous results showing that processing of synaptic cell adhesion molecules such as neuroligin1 by ADAM10 correlates with efficient synaptic transmission [67],[68]. These results suggest that ADAM10-mediated activity-dependent cleavage underlies the LTP alterations that we observe and also strongly argue that ADAM10 dependent ectodomain shedding of NG2 is a major contributory factor.

### Altered Composition of AMAPR: A Response to Decreased NMDAR Density in NG2^−/−^ Neurons

To gain insight into the synaptic mechanism leading to the LTP phenotype in the somatosensory cortex of NG2^−/−^ mice, we analyzed potential pre- and postsynaptic contributions. Basal synaptic transmission mediated by AMPARs was not altered at L4-to-L2/3 connections in NG2^−/−^ mice. This result, together with an unchanged short-term plasticity as addressed by paired-pulse facilitation of excitatory synaptic transmission, makes impairments of glutamate release rather unlikely. Nevertheless, a lack of LTP induction is often associated with reduced density or changed subunit composition of glutamatergic receptors at these neuronal synapses [69],[70]. In response to a constant stimulus strength, both NMDAR- and AMPAR currents were significantly reduced in L2/3 pyramidal neurons from NG2^−/−^ mice compared to WT controls. However, alterations of NMDAR subunit composition could be excluded as the respective kinetics and the current voltage relationship remained unaffected by NG2 deficiency, hence suggesting a down-regulation of NMDARs per se.

In contrast, AMPAR currents revealed remarkable differences between neurons of NG2^−/−^ and WT mice, including shorter decay time constants and an inward rectification of their *I-V* curve at positive holding potentials. The latter is characteristic for CP AMPARs lacking the GluR2 subunit, where it is caused by a polyamine-dependent block [71],[72]. Application of two selective antagonists, NASPM (GluR2 lacking AMPAR) and GYKI53655 (total AMPAR), confirmed the functional expression of these ion channels at excitatory synaptic inputs onto L2/3 pyramidal neurons of NG2^−/−^ mice. The presence of CP-AMPAR can explain the observed slightly hyperpolarized resting potential in the NG2^−/−^ neurons (Figure S2), since a more hyperpolarized resting potential has been described previously in neurons expressing CP-AMPAR [71]. CP-AMPARs are normally down-regulated during development in cortical pyramidal neurons, but retained in certain types of GABAergic interneurons in the adult brain [73],[74]. Here, similar to our studies, the insertion of CP-AMPARs is negatively correlated with the functional expression of CP-NMDARs in the postsynaptic membrane [75],[76], which influences the increase of intracellular Ca^2+^ and hence affects information processing within the somatosensory cortex.

Our results thus show that shedding of the NG2 ectodomain regulated by neuronal activity has a profound impact on neuronal glutamate receptor currents and long-term synaptic plasticity in the somatosensory cortex.

### The LNS Domains of the NG2 Ectodomain Restore the Impaired AMPAR Currents in NG2^−/−^ Neurons

Strikingly, the observed inward rectification in the *I-V* relationship at positive holding potentials in NG2^−/−^ mice could be restored to the WT phenotype by application of the N-terminal LNS domains of the NG2 ectodomain. In support of this, pre-incubation of cortical slices with the NG2 LNS domains prior to brief stimulation with glutamate increased activity-dependent neuronal c-fos expression in somatosensory neurons. These results demonstrate a direct influence of these specific domains [27] on the functional properties of AMPARs discussed above. Pyramidal neurons of NG2^−/−^ mice have more functional CP-AMPAR at postsynaptic membranes, which supports the concept of a stabilizing effect of the NG2-LNS domains on AMPAR containing the GluR2 subunit, which are impermeable to calcium. The LNS domains have been postulated by *in silico* experiments to belong to the same superfamily as pentraxins [77],[78]. Neuronal pentraxins such as Nptx2 (Narp) have been shown to bind to and stabilize GluR2 subunit-containing AMPAR at the neuronal plasma membrane [79]. Furthermore, application of the single LNS domain of β-neurexin 1 or β-neurexin 3, respectively, is sufficient to restore heterologous synapse formation in knock-out animals for each of these neurexins [29]. An alternative splicing variant of α-neurexin 3, resulting in an altered structure of one specific LNS domain, has recently been shown to impair postsynaptic AMPAR currents and LTP when introduced genetically [28].

The rescue in our experiments by recombinant NG2 LNS domains shows that these can diffuse within the slice to reach the sites of action on neurons. It is thus likely that endogenous NG2 ectodomain (containing these LNS domains) exhibits a limited diffusion within the ECM in vivo. The major 290 kD form of the soluble (in detergent-free tissue extract) NG2 ectodomain found by us and others is most likely mediating the endogenous NG2 signaling effects on neurons.

### Sensorimotor-Gating Behavior Is Impaired in NG2^−/−^ Mice

We observed altered NMDAR and AMPAR properties of L2/3 neurons of the somatosensory system in NG2^−/−^ mice. Functional integrity of the motorcortex (which is adjacent to the somatosensory cortex) was tested by two motor tests, Rotarod and CatWalk, and revealed no differences between the genotypes. A test assessing olfaction showed a reduced response to defined odors in the NG2^−/−^ mice, which could reflect a changed integration of sensory input in the olfactory cortex, as part of the somatosensory cortex. NG2^−/−^ mice also show impaired PPI in behavioral assays. Reduced PPI is associated with human diseases, such as schizophrenia, Huntington disease, Tourette syndrome, and obsessive-compulsive disorder [46]—diseases associated with an impaired integration of sensory data. Furthermore, receptors for three different neurotransmitters have been shown by pharmacological studies to participate in a reduction of PPI in rodents: stimulation of dopamine or serotonin receptors, or inhibition of NMDAR [46],[80],[81]. Two widely used learning and memory tasks (contextual fear conditioning and Morris Water Maze) showed no differences between NG2^−/−^ and WT control animals. These results suggest that the observed NG2-dependent alterations of the neuronal system affect in particular the neuronal networks of the somatosensory cortex (as tested by PPI), rather than those of the hippocampus-amygdala circuits (as tested by contextual fear conditioning and Morris Water Maze). This issue has to be addressed by further behavioral and molecular studies, to reveal if neuronal network activity is most strongly affected in the somatosensory cortex by the reported synaptic phenotype in NG2^−/−^ mice. We propose that synaptic signaling of the NG2 ectodomain influencing AMPAR/NMDR and LTP is likely to occur throughout the brain, as NG2 shedding on OPC seems to be a general phenomenon. On a molecular level, this could mean that in other areas of the brain such as the hippocampus, other regulators modulate LTP more strongly than the NG2 ectodomain.

Taken together, our data provide compelling evidence that OPCs in the adult brain are functionally integrated in the neuronal network and not only respond to but modulate neuronal activity via newly identified mechanisms. We show that NG2 is cleaved by ADAM10 and the γ-secretase on OPC. Furthermore, cleavage by ADAM10 releases the NG2 ectodomain into the ECM in an activity-dependent mechanism, where it influences postsynaptic glutamate receptor currents and neuronal LTP in the somatosensory cortex. We identify the LNS domains of the NG2 ectodomain as potent modulators of neuronal synapses, as they rapidly restore the altered AMPAR kinetics observed in the NG2^−/−^ mice to the WT pattern. These changes in sensorimotor processing are likely to underlie the behavioral alterations exhibited by NG2^−/−^ mice. Our results demonstrate a bidirectional cross-talk between OPC and the surrounding neuronal network, and demonstrate a novel physiological role for NG2-expressing OPC in regulating information processing. They also suggest a possible approach for modulating neuronal network function in disease.

## Materials and Methods

### Cell Cultures

The OPC cell line Oli-neu was cultured as described [38]. HEK 293 (Invitrogen) and MEF (PS1/2 KO, kind gift of C. Pietrzik) cells were cultured as described in Text S1. Cell lines were transfected with Lipofectamin2000 (Invitrogen) or polyethylenimine ([PEI], Sigma). Primary OPC (pOPC) were isolated from total brain of postnatal day 8–9 mice by magnetic cell sorting (Miltenyi) based on [82]. After 1 day in culture the sorted cell population consists of 90%–95% pure OPC [82].

### Cell Lysis and Fragmentation

Cells were lysed in Triton X-100, after removal of nuclei by centrifugation, supernatants were defined as PN lysates. Alternatively cells were homogenized without detergent with a Potter S teflon pestle, nuclei were removed by centrifugation, and after high-speed centrifugation fractions were defined as cytoplasmic, or crude membrane (CM) pellet fractions. For the cell free protease assays, CMs were obtained and incubated with different combinations of specific protease inhibitors (GI254023X [GI], GW280264X [GW]) [83]; DMSO and DAPT (Sigma), β-secretase inhibitor II (Calbiochem), Roche complete (PI) (Roche). After ultracentrifugation, supernatant (S100) and pellet (P100) were taken for Western blot.

### SDS-PAGE and Western Blotting

Equal volumes or amounts of total protein were separated on 4%–12% NuPage BisTris gradient gels (Invitrogen). Proteins were blotted on a PVDF membrane (Millipore). HRP-conjugated secondary antibodies were used with hyper films (GE) for detection. Protein levels were normalized against those of GAPDH.

### NG2^−/−^ Mice

Homozygous NG2-EYFP mice, (NG2^−/−^), lack NG2 protein expression and were previously described [42]. All experiments were carried out in strict accordance with protocols approved by local Animal Care and Use Committee of Johannes Gutenberg University of Mainz.

### LNS-AP and AP Purification

The two N-terminal LNS domains of NG2 (amino acids 30–382) were cloned in the pAPtag-5 (GenHunter) plasmid with a C-terminal AP, a Myc and hexaHis Tag; the construct was named LNS-AP. The original plasmid coding only for AP with Myc hexaHis Tag was used as control (AP). Proteins were purified from the cell and serum free medium of HEK over a nickel column (Sigma). Proteins were pre-incubated for 1 h (10 µg/ml) with acute slices for electrophysiology or immunocytochemistry (c-fos).

### Chemical Induced Activity in Acute Slices

Acute rat hippocampal slices [84] were incubated for 15 min with PFR or 4AP + BCC and subsequently transferred to standard artificial cerebrospinal fluid (aCSF) without Mg^2+^ and protease inhibitors, followed by tissue digestion with chondroitinase ABC [32] and Western Blot analysis.

### Electrophysiology

NG2^−/−^ mice and WT C57BL/6J mice (P22–P30) were used. Coronal slices (300 µm), containing somatosensory cortex were prepared in standard artificial cerebrospinal fluid (aCSF) solution, containing (mM): 125 NaCl, 2.5 KCl, 1.5 MgCl_2_, 2 CaCl_2_, 25 glucose, 25 NaHCO_3_, 1.25 NaH_2_PO_4_, bubbled with 95% O_2_/5% CO_2_ mixture (pH 7.4 at 37°C). Whole-cell patch-clamp recordings were performed in layer 2/3 pyramidal neurons in the somatosensory cortex visually controlled by DIC optics.

### Activity-Dependent Neuronal Gene-Expression

For investigation of c-fos expression in acute slices of the somatosensory cortex, slices were stimulated with glutamate (1 mM) for 10 min after pre-incubation (1 h) with purified LNS-AP or AP protein (10 µg/ml each). Experimental conditions (LNS-AP and AP) were compared from the same individual mouse and of a total of seven different mice were analyzed.

### Behavioral Tests

All behavioral tests were performed with the same cohort of mice between 5–8 weeks of age. Only male littermates were used (11 NG2^−/−^ and 10 WT animals). The tests performed were: Rota-Rod, CatWalk, test for olfaction, PPI, fear conditioning, Morris Water Maze.

### Statistics

Statistical analysis was done by using SPSS or MS Excel. Data were tested for normal distribution with non-parametric one-sample Kolmogorov-Smirnov test. All quantitative data are expressed as mean ± standard error of the mean (SEM). Significance was classified as follows: *, *p*≤0.05; **, *p*<0.01; ***, *p*<0.001; n.s. *p*>0.05. Classified by Student's *t* test or ANOVA with Dunnett's Multiple Comparison test.

For detailed description of methods refer to Text S1.

## Supporting Information

Figure S1 **NG2 ectodomain and LNS constructs.** (A) Immunoblot of saline (S1) and Triton X-100 (T1, membrane bound/associated proteins) soluble proteins from forebrains of adult WT and NG2^−/−^ mice. The T1 fraction contains all membrane bound/associated forms of NG2 including the FL protein (300 kD) and a small fraction of the ectodomain. In the S1 fraction the 290 kD ectodomain was detected as a major fragment, in the T1 fraction additionally a 275 and a 190 kD fragment and the FL protein were detectable. Full length NG2 (dashed rectangle) is exclusively present in the T1 fraction, detected by the NG2 cyto AB, which shows no band at 300 kD in the NG2^−/−^. (B) Schematic illustration of the NG2 protein and the LNS-AP (105 kD) and AP (62 kD) recombinant proteins. LNS-AP consists of the two N-terminal LNS domains of the NG2 ectodomain, with a C-terminal AP, Myc, and a hexa-His Tag. AP with Myc and hexa-His Tag was used as a control. Coomassie stained SDS-PAGE gel shows the purity of eluted proteins from the nickel-columns used to purify the recombinant proteins. Western Blot of OPC cell lysate and the isolated proteins shows immunoreactivity with an anti hexa-His antibody for both constructs. NG2 ectodomain specific antibody (pc AB NG2) shows specific bands only for the LNS-AP construct and FL NG2 protein from the total OPC cell-lysate.
(TIF)

Figure S2 **Altered membrane potential of L2/3 pyramidal neurons from NG2^−/−^ mice.** (A) Example traces of voltage responses elicited by depolarizing current steps at −100 to 400 pA. Cells were current clamped to −70 mV. (B–D) Intrinsic membrane properties of L2/3 pyramidal neurons illustrated by resting membrane potential (B), membrane resistance (C), and membrane capacitance (D). Unpaired Student's *t* test (*p<0.05; n.s. *p*> 0.05). Data represent the mean ± SEM.
(TIF)

Figure S3 **sEPSCs remain unchanged after NG2 deletion.** (A) Example traces of AMPAR-mediated sEPSCs recorded at −80 mV. Application of AMPAR blocker DNQX (20 µM) verified AMPAR dependent currents (bottom traces). (B, C) Mean frequency and amplitude of sEPSCs remained unchanged in pyramidal cells from NG2^−/−^ mice. Unpaired Student's *t* test (n.s. *p*>0.05). (D, E) Summary plots showing rise time (D) and decay time (E) of sEPSCs. Unpaired Student's *t* test (n.s. *p*>0.05). Data represent the mean ± SEM. (F) Input-output relation of AMPAR-mediated EPSCs recorded at −80 mV. Unpaired Student's *t* test (***p<0.001). Data represent the mean ± SEM.
(TIF)

Figure S4 **Paired pulse ratio of evoked EPSCs are unaffected in NG2^−/−^ mice.** (A) Representative EPSCs traces evoked by a paired stimulus with interevent interval (ISI) of 30 ms. (B) Summary plot of the paired-pulse ratio (ratio of the 2nd to 1st EPSC), taken at distinct interstimulus intervals (ISI), shows no differences between NG2^−/−^ and WT groups.
(TIF)

Figure S5 **Coomassie gel used for normalization.** Levels of ECM-associated NG2 ectodomain were determined by Western blot in the supernatant resulting from chondroitinase ABC digestion of acute slices (Figure 3C and 3D). (A) Normalization was performed by comparing the corresponding lanes of the NG2 Western blot with the matching lanes on the coomassie stained gel. (B) Normalization of the total supernatant protein identified by coomassie staining is shown for duplicates from one experiment.
(TIF)

Figure S6 **Protease assay.** The same experimental setup was used as in Figure 2A. (A) The monoclonal antibody reacting with an epitope in the NG2 extracellular domain (NG2 mc AB), detected bands of around 300 kD on a Western blot of the P100 and S100 fraction. In contrast, an antibody recognizing the ICD (NG2 cyto AB) only detected one major band in P100 but did not react with proteins of around 300 kD in the S100 fraction. (The lane marker 2 contains hand-drawn lines on the film marking the positions of the MW markers). (B) An inhibitor of the β-secretase (β-secretase inhibitor II, 25 µM) had no effect on levels of the protein band of around 300 kD in the S100 fraction. In contrast, α-secretase inhibitors (GI, GW) caused a decrease in levels of the protein band of around 300 kD in comparison to the control (PI 1×).
(TIF)

Data S1 **Rawdata.**
(XLSX)

Text S1 **Supplemental materials and methods.**
(DOC)

## Abbreviations

4-AP: 4-aminopyridine
AMPA: α-amino-3-hydroxy-5-methyl-4-isoxazolepr opionicacid
AMPAR: AMPA receptor
AP: alkaline phosphatase
APP: amyloid precursor protein
BCC: bicuculline
CNS: central nervous system
CP: Ca^2+^-permeable
CTF: C-terminal fragment
ECM: extracellular matrix
EPSP: excitatory post-synaptic potentiation
EYFP: enhanced yellow fluorescent protein
FL: full length
Glut: glutamate
HEK: human embryonic kidney 293
ICD: intracellular domain
LNS: lamininG-neurexin-sex hormone binding globulin
(c)LTP: (chemical) long-term potentiation
MEF: mouse embryonic fibroblast
NG2: nerve-glia antigen 2
NMDA: N-methyl-D-aspartate
NMDAR: NMDA receptor
OPC: oligodendrocyte precursor cells
PFR: picrotoxin, forskolin, rolipram
PN: post nuclear
PPI: prepulse inhibition
SEM: standard error of the mean
sEPSC: spontaneous excitatory postsynaptic current
TBS: theta-burst stimulus
WT: wild-type
