# Supplementary figures and images for: Oligodendrocyte Precursor Cells Modulate the Neuronal Network by Activity-Dependent Ectodomain Cleavage of Glial NG2
Source: PLoS Biol. 2014 Nov 11;12(11):e1001993. doi: 10.1371/journal.pbio.1001993 (PMC4227637; doi:10.1371/journal.pbio.1001993)

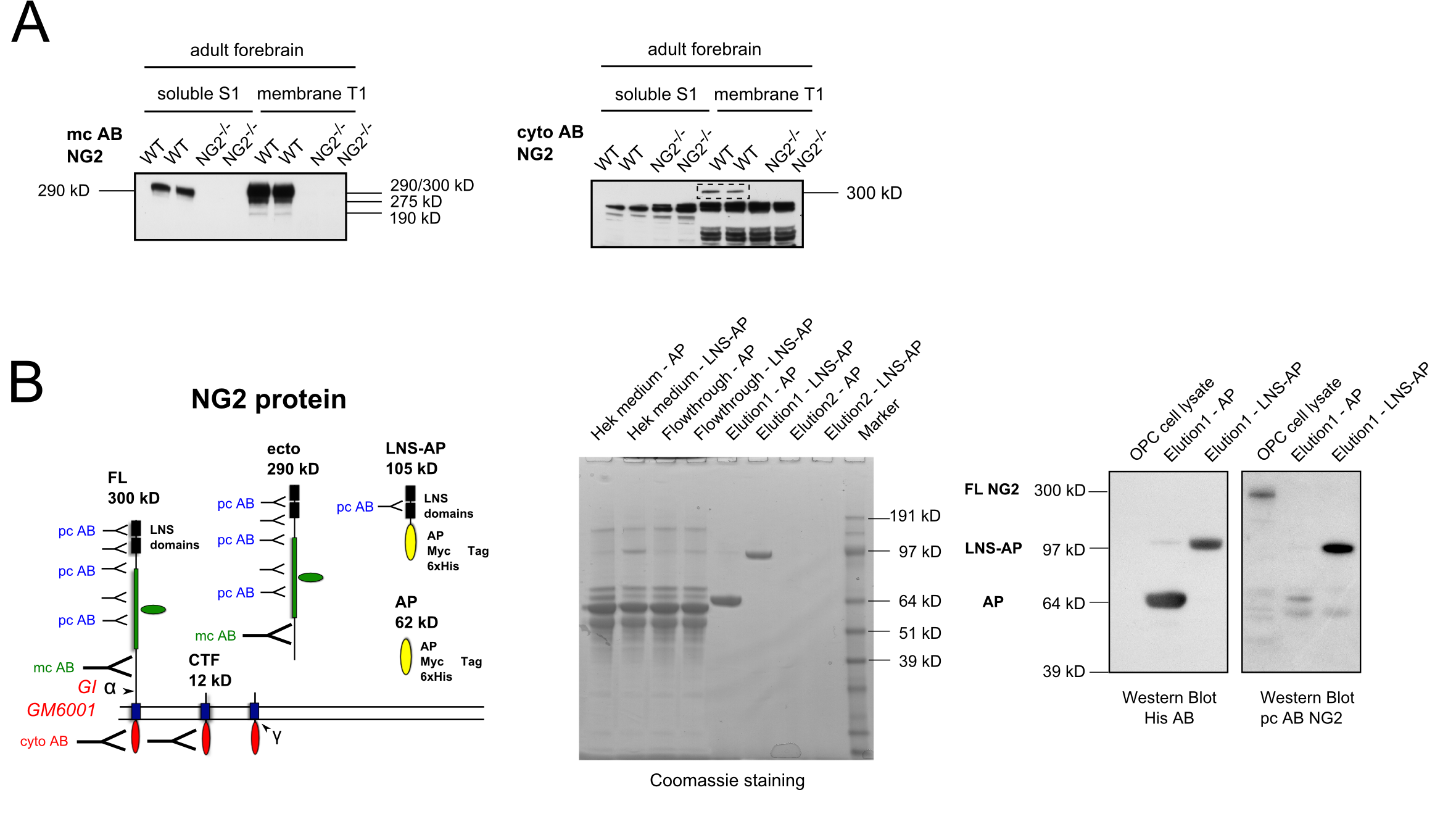

Supplement: Figure S1 — NG2 ectodomain and LNS constructs. (A) Immunoblot of saline (S1) and Triton X-100 (T1, membrane bound/associated proteins) soluble proteins from forebrains of adult WT and NG2−/− mice. The T1 fraction contains all membrane bound/associated forms of NG2 including the FL protein (300 kD) and a small fraction of the ectodomain. In the S1 fraction the 290 kD ectodomain was detected as a major fragment, in the T1 fraction additionally a 275 and a 190 kD fragment and the FL protein were detectable. Full length NG2 (dashed rectangle) is exclusively present in the T1 fraction, detected by the NG2 cyto AB, which shows no band at 300 kD in the NG2−/−. (B) Schematic illustration of the NG2 protein and the LNS-AP (105 kD) and AP (62 kD) recombinant proteins. LNS-AP consists of the two N-terminal LNS domains of the NG2 ectodomain, with a C-terminal AP, Myc, and a hexa-His Tag. AP with Myc and hexa-His Tag was used as a control. Coomassie stained SDS-PAGE gel shows the purity of eluted proteins from the nickel-columns used to purify the recombinant proteins. Western Blot of OPC cell lysate and the isolated proteins shows immunoreactivity with an anti hexa-His antibody for both constructs. NG2 ectodomain specific antibody (pc AB NG2) shows specific bands only for the LNS-AP construct and FL NG2 protein from the total OPC cell-lysate. (TIF) [file pbio.1001993.s001.tif]

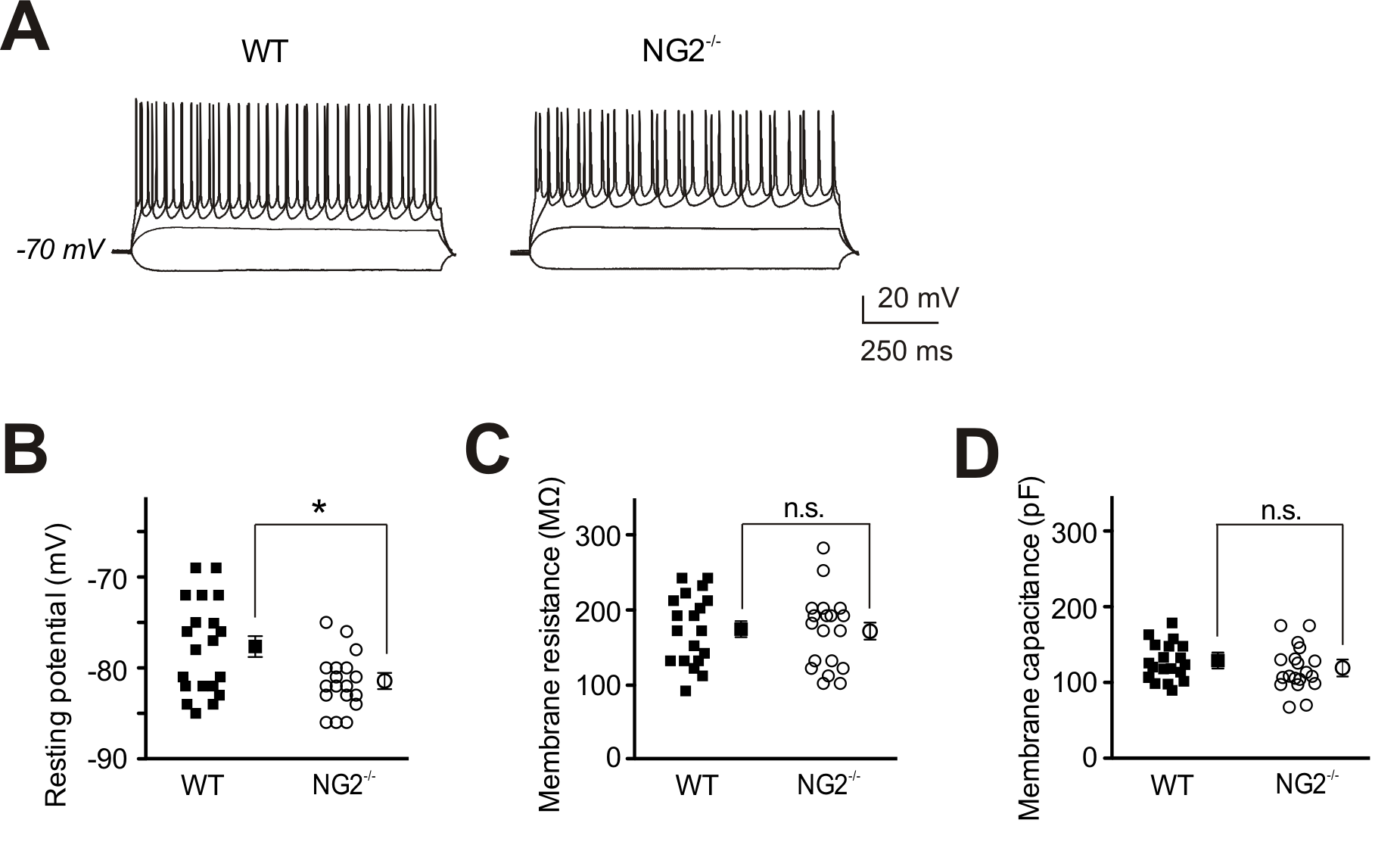

Supplement: Figure S2 — Altered membrane potential of L2/3 pyramidal neurons from NG2−/− mice. (A) Example traces of voltage responses elicited by depolarizing current steps at −100 to 400 pA. Cells were current clamped to −70 mV. (B–D) Intrinsic membrane properties of L2/3 pyramidal neurons illustrated by resting membrane potential (B), membrane resistance (C), and membrane capacitance (D). Unpaired Student's t test (*p<0.05; n.s. p> 0.05). Data represent the mean ± SEM. (TIF) [file pbio.1001993.s002.tif]

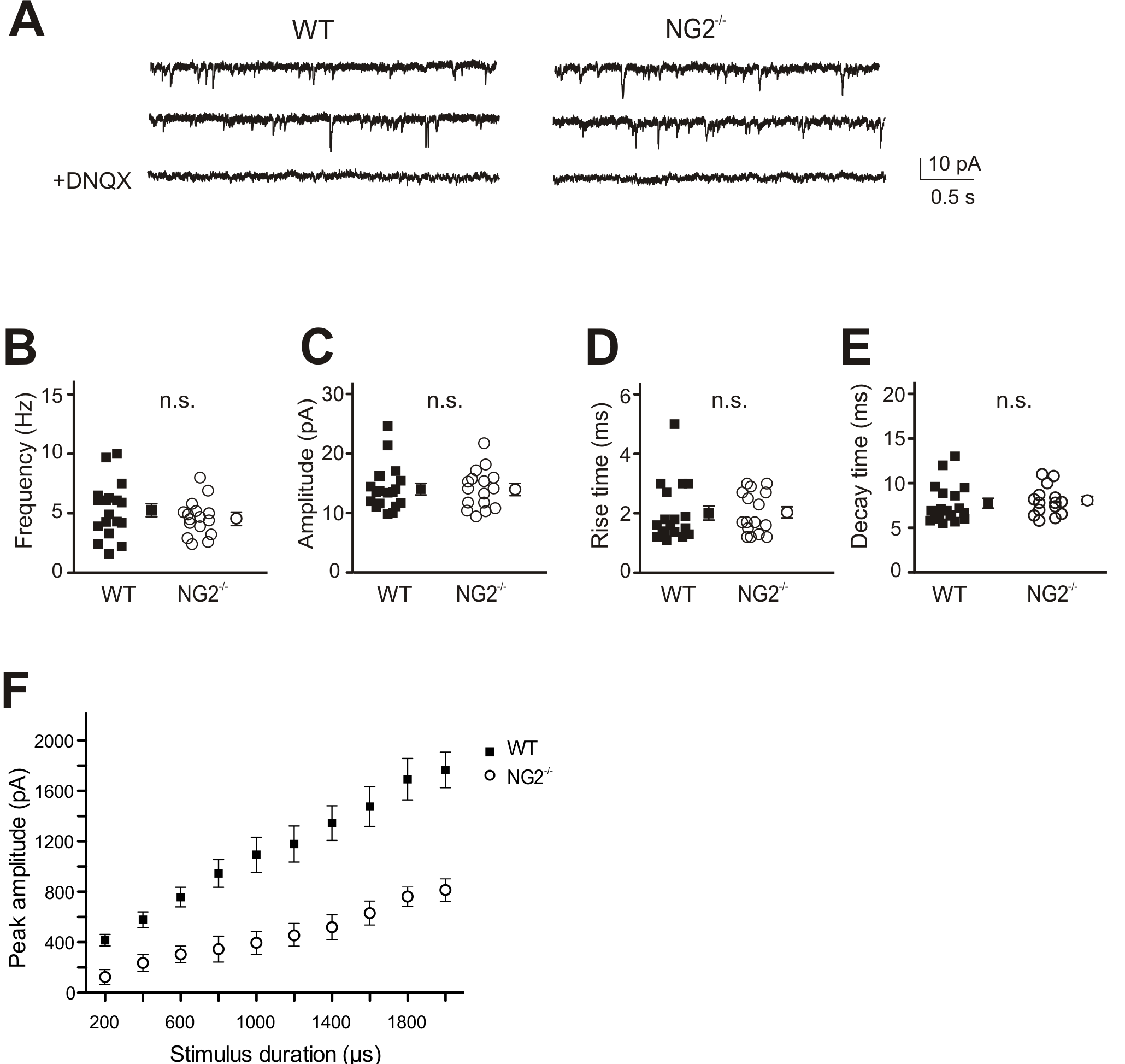

Supplement: Figure S3 — sEPSCs remain unchanged after NG2 deletion. (A) Example traces of AMPAR-mediated sEPSCs recorded at −80 mV. Application of AMPAR blocker DNQX (20 µM) verified AMPAR dependent currents (bottom traces). (B, C) Mean frequency and amplitude of sEPSCs remained unchanged in pyramidal cells from NG2−/− mice. Unpaired Student's t test (n.s. p>0.05). (D, E) Summary plots showing rise time (D) and decay time (E) of sEPSCs. Unpaired Student's t test (n.s. p>0.05). Data represent the mean ± SEM. (F) Input-output relation of AMPAR-mediated EPSCs recorded at −80 mV. Unpaired Student's t test (***p<0.001). Data represent the mean ± SEM. (TIF) [file pbio.1001993.s003.tif]

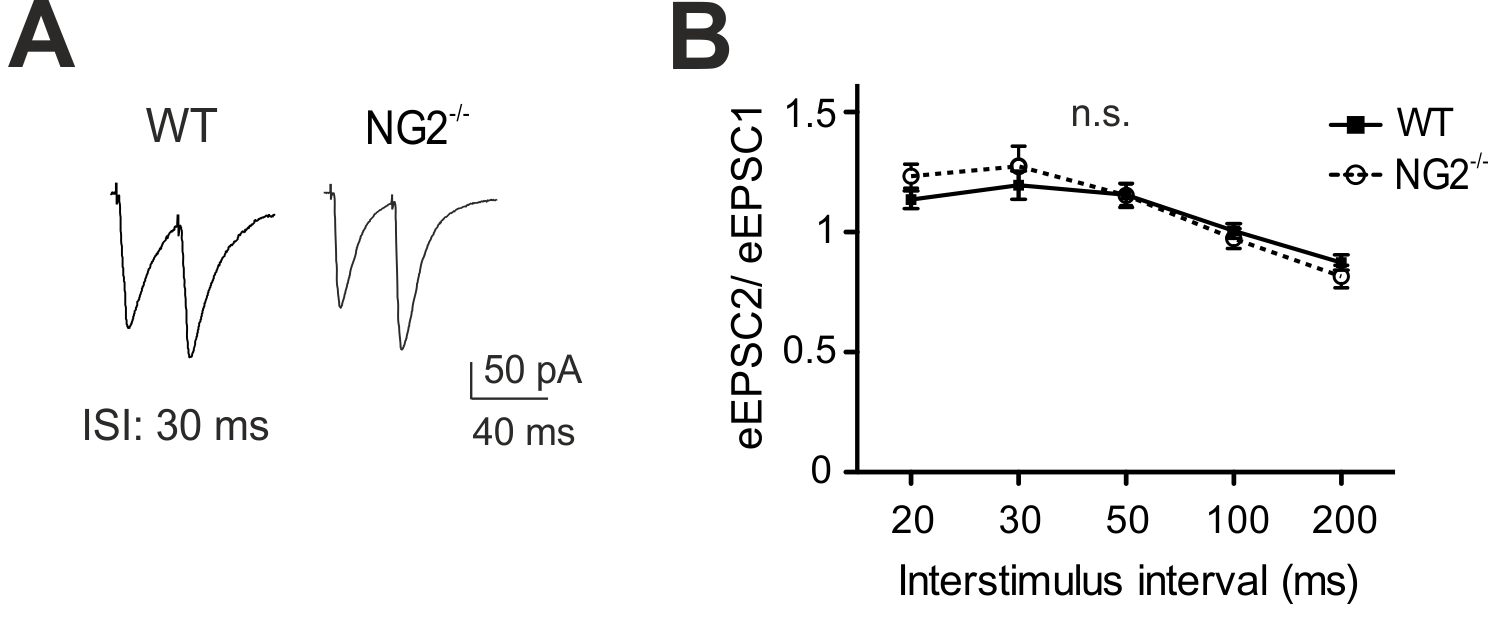

Supplement: Figure S4 — Paired pulse ratio of evoked EPSCs are unaffected in NG2−/− mice. (A) Representative EPSCs traces evoked by a paired stimulus with interevent interval (ISI) of 30 ms. (B) Summary plot of the paired-pulse ratio (ratio of the 2nd to 1st EPSC), taken at distinct interstimulus intervals (ISI), shows no differences between NG2−/− and WT groups. (TIF) [file pbio.1001993.s004.tif]

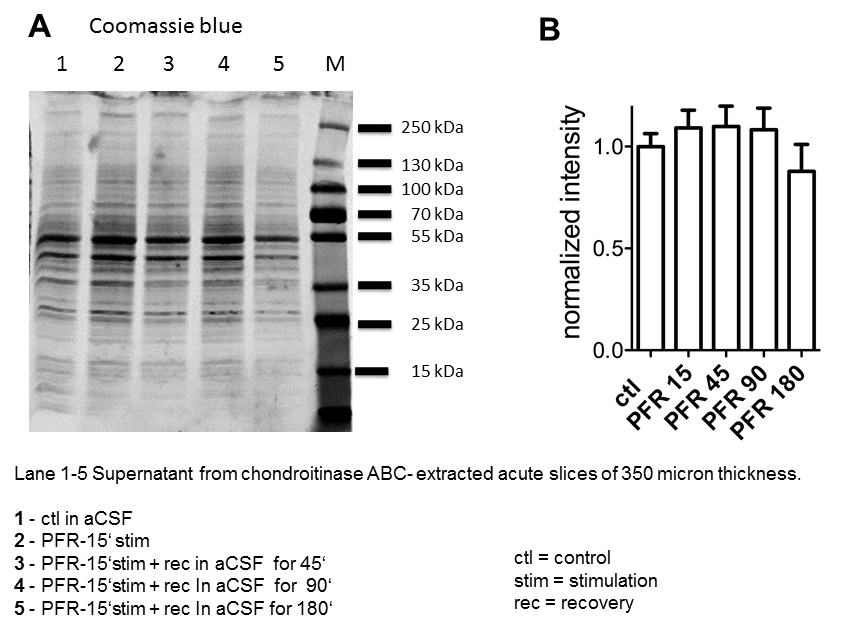

Supplement: Figure S5 — Coomassie gel used for normalization. Levels of ECM-associated NG2 ectodomain were determined by Western blot in the supernatant resulting from chondroitinase ABC digestion of acute slices (Figure 3C and 3D). (A) Normalization was performed by comparing the corresponding lanes of the NG2 Western blot with the matching lanes on the coomassie stained gel. (B) Normalization of the total supernatant protein identified by coomassie staining is shown for duplicates from one experiment. (TIF) [file pbio.1001993.s005.tif]

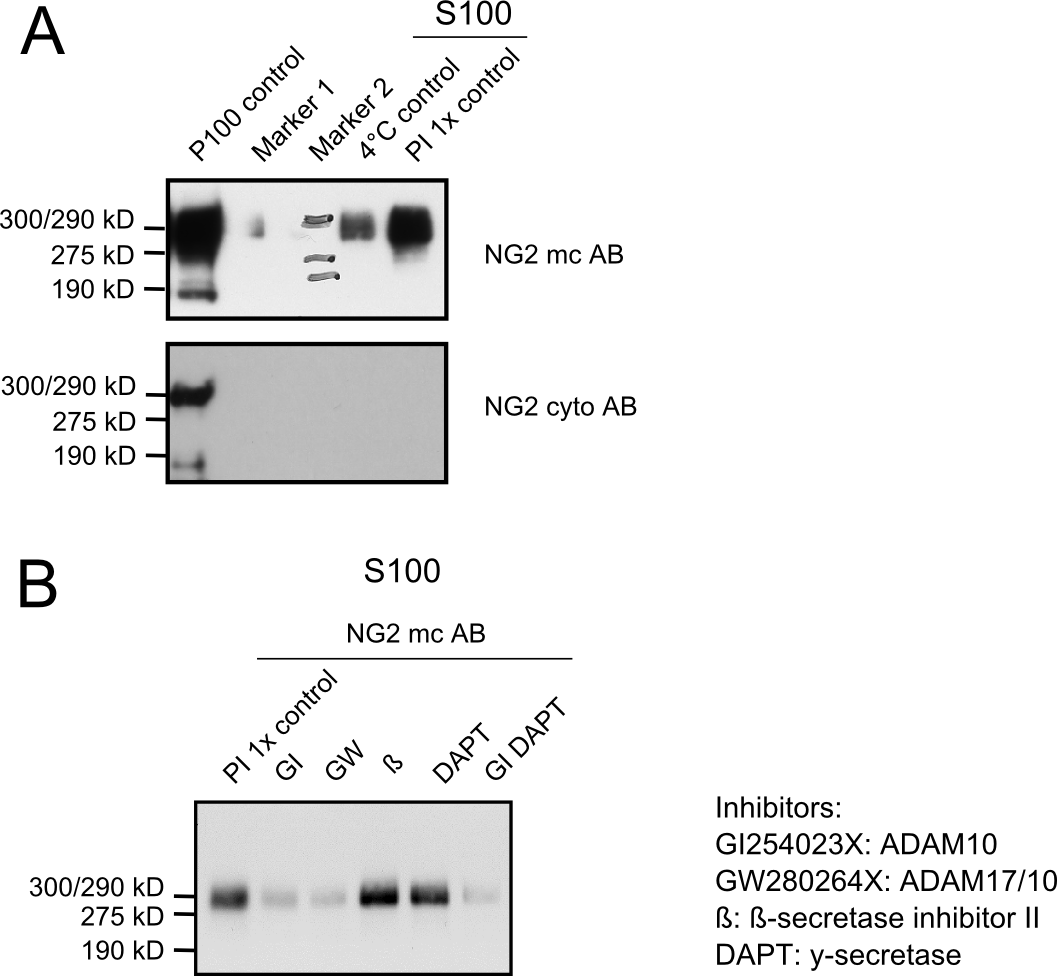

Supplement: Figure S6 — Protease assay. The same experimental setup was used as in Figure 2A. (A) The monoclonal antibody reacting with an epitope in the NG2 extracellular domain (NG2 mc AB), detected bands of around 300 kD on a Western blot of the P100 and S100 fraction. In contrast, an antibody recognizing the ICD (NG2 cyto AB) only detected one major band in P100 but did not react with proteins of around 300 kD in the S100 fraction. (The lane marker 2 contains hand-drawn lines on the film marking the positions of the MW markers). (B) An inhibitor of the β-secretase (β-secretase inhibitor II, 25 µM) had no effect on levels of the protein band of around 300 kD in the S100 fraction. In contrast, α-secretase inhibitors (GI, GW) caused a decrease in levels of the protein band of around 300 kD in comparison to the control (PI 1×). (TIF) [file pbio.1001993.s006.tif]
