# Supplementary material for: Oligodendrocyte Precursor Cells Modulate the Neuronal Network by Activity-Dependent Ectodomain Cleavage of Glial NG2
Source: PLoS Biol. 2014 Nov 11;12(11):e1001993. doi: 10.1371/journal.pbio.1001993 (PMC4227637; doi:10.1371/journal.pbio.1001993)
Supplement: Text S1 — Supplemental materials and methods. (DOC) [file pbio.1001993.s008.doc]

**Supplemental Materials & Methods**

*Cell lines*

The OPC cell line Oli-neu was cultured on poly-L-Lysine (PLL) coated dishes in SATO medium with 1% Horse Serum (HS) . Human embryonic kidney cells (HEK293, HEK) and mouse embryonic fibroblasts (MEF) lacking presenilin 1 and 2 (PS1/2 KO, kind gift of Prof. C. Pietrzik) were cultured in Dulbecco's modified Eagle's medium (DMEM, Sigma) with 1% pyruvate and 10% fetal calf serum (FCS). The ADAM10 inhibitor GI was incubated for 6 hours (5 µM), following subsequent incubation of 12 hours with (2.5 µM). DAPT was used at 5-10 µM for the stated periods of time. All secretase inhibitors were added directly to culture medium, the control was treated with vehicle alone (DMSO).

*Transient transfection of cell lines*

MEF cells were transfected with Lipofectamin2000 (Invitrogen). HEK and OPC cell-line were transfected using polyethylenimine (PEI, 1 µg/µl, Sigma) at a ratio of 1:4 (DNA:PEI; w/v). Transfection solutions were applied to cells after 16h of culture. Cell lysates were analyzed 48 hours after transfection.

The following plasmids were used: ADAM10 (pcDNA6, human cDNA, kind gift of Dr. R. Postina); ADAM17HA (pcDNA3, bovine cDNA, ; NG2_del (pRK5, mouse cDNA, AS 1-477_Δ_2206-2327); NG2_ICD (pRK5, mouse cDNA, AS 2251-2327); NG2_del_Myc (pcDNA4); NG2_ICD_Myc (pcDNA4); Flag_NG2_del (pIRESHyg2).

*Stimulation of primary OPC isolated by magnetic cell sorting*

Primary OPC (pOPC) were isolated from total brain of postnatal day 8-9 mice by magnetic cell sorting (Miltenyi) based on . After one day in culture the sorted cell population consists of 90-95% pure OPC (NG2+ and Olig2+ cells and no pericytes SMA+ and NG2+ cells) (, Sakry et al., unpublished data). For short time stimulations, the chemicals were directly added to the culture medium: picrotoxin 50µM, forskolin 50µM, and rolipram 0.1µM (PFR) or 4-aminopyridine 2.5mM and bicuculline 50µM (4AP+BCC) for 20 min and glutamate 1 mM for 10 min. The NMDAR inhibitor (MK801) or the ADAM10 inhibitor (GI) (each 5 µM) were pre-incubated for 15 min before the stimulations.

*Postnuclear cell lysates*

Cells were washed with PBS and scraped with a rubber policeman in lysis buffer (PBS, 1% Triton X-100, protease inhibitor cocktail, Roche complete) from the culture plate. After incubation for 30 min at 4°C and centrifugation at 1000 x g, 10 min, 4°C, supernatants were defined as postnuclear (PN) lysates. The same volume of lysis buffer was used per sample and loaded for WB.

*Crude membrane, cytoplasmic and nuclear fractionation*

Confluent cells from 15 cm culture dishes were washed and suspended in PBS. Cells were pelleted with 133 x g, 10min, 4°C and dissolved in homogenization buffer (10 mM Mops, 10 mM KCl, pH 7.0) with protease inhibitors (PI). Homogenization was done with a Potter S teflon pestle (20 strokes, 900rpm), nuclei were then pelleted at 1000 x g, 15 min, 4°C. The supernatant was centrifuged at 16.000 x g, 40 min, 4°C resulting in a crude membrane (CM) fraction and a cytoplasmic (CP) fraction. The nuclear fraction was resuspended twice in homogenization buffer and triturated 10x with a 24G gauge needle. CM and nuclear fractions were dissolved in 4x SDS sample buffer, the CP fraction was diluted into 2x SDS sample buffer to the same final volume. Equivalent volumes were loaded for WB.

*In vitro protease assays*

Crude membranes (CM) were obtained as previously described. The membrane fraction was resuspended in 100 µl (per 15 cm dish) reaction buffer (150 mM sodium citrat, pH 6.4). After 10 min incubation on ice, 50 µl of CM were incubated for 2 hours, 37°C with different combinations of specific protease inhibitors. The general protease inhibitor (1xPI, Roche complete), was present in every reaction. Subsequently ultracentrifugation was performed (100.000 x g, 1 hour, 4°C). Complete supernatant and the dissolved pellet of one reaction were used for WB. Protease inhibitors: GI254023X (GI), GW280264X (GW) ; DMSO and DAPT Sigma; PI = protease inhibitor, Roche complete without EDTA; if inhibitors were used in cell culture, they were diluted in the culture medium.

*LNS-AP and AP purification*

The two N-terminal LNS domains of NG2 (amino acids 30-382) were cloned in the pAPtag-5 (GenHunter) plasmid with a C-terminal alkaline phosphatase (AP), a Myc and hexa-His Tag, the construct was named LNS-AP. The original plasmid coding only for AP with Myc hexa-His Tag was used as control (AP). The N-terminal endogenous membrane localization sequence (amino acids 1-29) was replaced by a secretion sequence already included in the plasmid. HEK cells were transfected with LNS-AP or AP using polyethylenimine (PEI) (see above). 6 hours after transfection the medium was changed to serum-free medium and this was collected 24 hours after transfection. His tagged proteins were purified from the cell free medium using a nickel column (Sigma). After elution with imidazole, purified proteins were dialyzed against Ringer solution. Protein concentration and purity were examined by SDS-PAGE followed by Coomassie staining, blotting with antibodies against NG2 and measurement of absorption at 224 nm.

*SDS-PAGE and Western Blotting*

Equal volumes of protein solution or amounts of total protein were used for analysis. Protein concentration was determined by the BCA protein assay (Pierce). All samples were diluted with 1-4x SDS or LDS (Invitrogen) sample buffer, heated to 80°C for 10 min and resolved on 4–12% NuPage BisTris gradient gel in combination with MES or MOPS running buffer (Invitrogen). Western blotting was done with the respective NuPage Blot system utilizing a polyvinylidene fluoride (PVDF) membrane (Millipore). The latter was blocked in PBS containing 4% nonfat milk and 0.1% Tween 20.

The following primary antibodies were used: NG2 mc, rat, 1:200 ; NG2 cyto, rabbit, 1:500 ; NG2 pc, rabbit, 1:750 ; GAPDH, rabbit, 1:2500 (Bethyl); Myc, rabbit, 1:500 (abcam); CT15, rabbit, 1:10.000 ; α-Tubulin, mouse, 1:10.000 (Sigma); ADAM10, rabbit, 1:500 (abcam); Flag, mouse, 1:500 (Sigma);

Signal detection was carried out using HRP-conjugated secondary antibodies 1:10.000 (Dianova), enhanced chemiluminescence (ECL) assay solution (Millipore) and hyper films (GE). ImageJ 1.46 (NIH) was used for signal quantification and. All protein levels were normalized against those of GAPDH from the same sample.

*NG2^-/-^ mice*

The NG2-EYFP mouse line on the C57BL/6N background has been previously described . The EYFP coding sequence is inserted into the first exon of the endogenous NG2 locus, which leads to the replacement of one NG2 allele by EYFP under the regulation of the endogenous NG2 promoter. In the homozygous state (NG2^-/-^EYFP^+/+^), NG2 expression is lacking.

*Chemical induced activity in acute slice cultures*

Acute hippocampal slices were prepared from 8-10 week old wistar rats as described previously . Acute hippocampal slices of 350 µm thickness were prepared using the Mcllwain tissue chopper (Mickle Laboratory) in ice cold artificial cerebrospinal fluid (aCSF: 125 mM NaCl, 2.5 mM KCl, 1.25 mM NaH_2_PO_4_, 25 mM NaHCO_3_, 2 mM CaCl_2_, 1 mM MgCl_2_, 25 mM glucose) and subsequently recovered for 45 minute at room temperature and 100 minutes at 32°C. During the whole procedure the solution was bubbled with 95% O_2_ and 5% CO_2_. Chemical stimulation with combination of 50 µM picrotoxin, 50 µM forskolin, and 0.1 µM rolipram (PFR) in ACSF with no Mg^2+^ was given for 15 min. After the treatment slices were collected in Tyrod’s buffer containing Chondroitinase ABC (0.1 M Tris-HCl, pH 8.0 containing 0.03 M sodium acetate) for digestion of CSPGs (chondroitin sulphate proteoglycan) as described . Enzymatic extraction was performed for 50-60 min at 32°C. Slices were then triturated and centrifuged at 12000 rpm and supernatants were collected for Western blotting and normalized against total protein from according Coomassie gel. Protease inhibitors were preincubated with slices for 30 min and were present for the whole experiment. GM6001 was used at 25 µM and GI254023X at 5 µM. Treatment with 2.5 mM 4-aminopyridine and 50µM bicuculline (4AP + BCC) for 10 min and subsequently 20 min recovery time was carried out under the same conditions. All acute slice stimulation experiments were performed at the Leibnitz Institute for Neurobiology (LIN, Magdeburg).

*Electrophysiology*

*NG2^-/-^* mice and wild-type (WT) C57BL/6N mice (P22 to P30) were anesthetized with isoflurane before decapitation. Coronal slices (300 µm) containing the somatosensory cortex were cut with a vibratome (Leica VT 1000S, Germany) in an ice-cold artificial cerebrospinal fluid (ACSF) solution, containing (mM): 125 NaCl, 2.5 KCl, 1.5 MgCl_2_, 2 CaCl_2_, 25 glucose, 25 NaHCO_3_, 1.25 NaH_2_PO_4_, constantly bubbled with 95% O_2_/5 %CO_2_ mixture (pH 7.4 at 37°C). Whole-cell recordings were made in layer 2/3 pyramidal neurons in the somatosensory cortex under visual control with DIC optics. *NG2^-/-^-EYFP* glia cells were visualized with a GFP (480 nm)-fluorescence filter. In voltage-clamp mode the recording pipettes (5-6 MΩ; GB150F-8P, Science Products) were either filled with an internal solution containing (mM): 125 CsGlu, 5 CsCl, 10 EGTA, 2 Na_2_-ATP, 2 MgCl_2_, 0.4 GTP, 10 HEPES, 5 QX-314 (pH 7.2) for all voltage-clamp recordings. In some experiments D-AP5 (25 µM) or DNQX (20 µM) was bath applied to isolate NMDAR- or AMPAR-mediated EPSCs, respectively. Current-clamp recordings were made with an internal solution containing (mM): 140 potassium gluconate, 2 MgCl_2_, 4 Na_2_-ATP, 0.3 Na_2_-GTP, 10 Na-phosphocreatin, 10 HEPES, 0.2 spermine (*in experiments indicated*) and 1mg/ml biocytin (pH 7.2). Electrophysiological signals were acquired using Axopatch 200B amplifier (Axon-Instruments, Union City, USA) and pClamp10 software (Molecular Devices, Sunnyvale, USA). Signals were low-pass filtered at 2 kHz and digitized at 10 kHz. Access resistance was constantly monitored. Cells exhibiting access resistance of > 20 MΩ or more than 20% during an experiment were discarded. *Intrinsic cell properties* were assessed in current-clamp mode in presence of synaptic blockers (DNQX, D-AP5, PTX) at a holding potential of -70 mV and in response to depolarizing current steps ranging from -100 to 400 pA (ΔI = 20 pA; 1 s). *Spontaneous EPSCs* (sEPSCs) were recorded for a minimum of 5 min at a holding potential of -80 mV and 10 min after establishing whole-cell configuration. AMPAR-mediated sEPSCs were verified with bath application of DNQX (20 µM). *Evoked EPSCs* (eEPSCs) were elicited in L2/3 pyramidal neurons by focal electrical stimulation through a glass pipette (5 MΩ) filled with ACSF and positioned on layer 4. Pulse duration was set to 0.05 ms. Pulse intensity was adjusted to obtain reliable monosynaptic response amplitudes. The stimulation-conditions were kept constant between WT- and NG2^-/-^ -slices, even though the peak amplitudes in the NG2^-/-^ group differed in comparison to WT. The *ratio of NMDAR- to AMPAR-mediated currents* was calculated from mean peak amplitude of AMPAR-mediated eEPSCs recorded at -80 mV and isolated NMDAR-mediated eEPSCs recorded at +60 mV in presence of 20 µM DNQX. The stimulation intensity was set to evoke an EPSC with a signal amplitude of 70-75% of maximum response, eliciting reliable AMPAR- and NMDAR-mediated EPSC responses in L2/3 pyramidal neurons from WT animals.

*Current-voltage (I-V) relationships* of NMDAR-mediated currents were studied by normalizing the stimulus intensity to a fixed AMPAR-mediated current amplitude. NMDAR-mediated currents were isolated by bath application of DNQX (20 mM) and evaluated at different holding potentials, ranging from -80 to 60 mV. *I-V* relations of AMPAR-mediated currents were studied in response to maximal stimulation intensity, at different holding potentials ranging from -80 mV to +40 mV, with V_m_ of 20 mV. Spermine (0.2 mM) was added to the intracellular solution to maintain rectification. *NMDAR-dependent LTP* was investigated in current-clamp mode and the membrane potential of all cells investigated was fixed to -80 mV. Single test pulses (0.05 ms) were delivered to L2 afferents at 0.033 Hz to induce subthreshold EPSPs, which did not vary more than 20 % during 10 minutes baseline recording and between the groups. A modified high-frequency theta-burst stimulation (*TBS*) contained 5 synaptic trains (at 20 s intervals) of 5 burst (at 5 Hz) each consisting of four stimuli at 100 Hz. Each TBS was paired with a depolarizing current step (1100 pA, 45 ms duration) delayed by 5 ms, aiming to induce dendritic spikes in all recorded neurons. Following TBS, single EPSPs were recorded at baseline stimulation for minimum of 30 minutes. Input resistance was continuously monitored by applying a negative current step of -30 pA through the patch pipette following each test pulse. Changes in the amplitude of EPSPs were analyzed during 20-30 min after TBS. Slices containing biocytin-filled pyramidal cells were PFA fixated and further immunohistochemically treated for morphological analysis.

*Solutions/ chemicals used in electrophysiology:* Picrotoxin (PTX, 50 µM), D-AP5 (25 µM), DNQX (20 µM), CGP55845 hydrochloride (1 µM) were obtained from Tocris/ Biozol (Eching, Germany). Spermine tetrahydrochloride (0.2 mM), QX-314 (5 mM) and 1-Naphthylacetyl spermine trihydrochloride (NASPM; 100 µM; 15 min incubation) and GYKI53655 (25 µM; 15 min incubation) derived from Sigma-Aldrich (Munich, Germany). GM6001 (20 µM; one hour pre-incubation and administration during LTP recordings) was purchased from Calbiochem.

*Data evaluation of detected signals:* Data were evaluated offline using pCLamp SoftwareSpontaneous events were identified automatically with Mini Analysis Software (Synaptosoft, USA), followed by validated by careful visual inspection. The threshold for event detection was set 2.5 times the root mean square noise level. The frequency and amplitude were calculated as the median from 600 events for all tested cells. The threshold for event detection was set 2.5 times root mean square noise level and subsequently verified by visual inspection. Evoked EPSCs were analyzed by using the average of 6-10 consecutive traces. The kinetics of AMPAR- and NMDAR- mediated EPSCs were determined at a membrane potential of -80 mV and +60 mV, respectively. The rise time of glutamatergic currents was calculated as the time of 10-90% onset of the peak amplitude. The decay time was calculated by fitting the decay of the current to a single-exponential function: *f(t)=A_i_e^-t/τ^_i_+C*. The rectification index (RI) was defined as [(the ratio of AMPAR conductances recorded at +40 and -60 mV) X 1.5], for correction of differences in the driving force **.** Input resistance was calculated according Ohm’s law in response to a hyperpolarizing step of -100 pA lasting 1 s.

*Immunohistochemistry staining of sections*

Primary antibodies used were: MAP-2 (rabbit) Abcam, SMI 31 (mouse) Covance, GFP (chicken) Abcam, NeuN (mouse) Millipore, c-fos (rabbit), Calbioche, DAPI (Sigma) was used to stain nuclei.

Secondary antibodies: Alexa488 goat anti-chicken Invitrogen, Cy3 goat anti-rabbit Dianova, Cy5 goat anti-mouse Dianova.

Brains of heterozygous NG2^-/-^EYFP^+/+^ mice were fixed for 2h in 4% paraformaldehyde (PFA) at 4 C°. Coronal sections with a thickness of 50 µm were cut with a Leica VT 1000S vibratome. Slices were permeablised and fixed for 2h at RT (PBS, 0.4 % TX-100, 10% normal goat serum (NGS)). First antibodies were applied for 24h at 4 C° (PBS, 10% NGS). PBS washing was applied 3 times for 15 min. Secondary antibodies were applied for 2h at RT (PBS, 10% NGS), followed by washing in PBS. Slices were mounted in Moviol on poly-L-lysine-coated slides.

For investigation of c-fos expression in the somatosensory cortex, individually vibratome-prepared acute slices of WT and NG2^-/-^ mice (of 300 µm thickness) were pre-incubated for 50 min in oxygenated ACSF solution containing PTX (50 µM) and 10 µg/ml LNS-AP or AP protein, followed by 10 min of incubation with 1mM glutamate. Slices were fixed in 4% PFA overnight at 4°C, subsequently thinner slices of 60 µm were cut and stained for immunohistochemistry as described above using NeuN (1:1000) and c-fos (1:1000) antibodies.

*Image acquisition and processing*

Low power images were taken with a Leica DM6000 deconvolution microscope and joined together with the metamorph imaging software. Confocal laser scanning microscope (CLSM) images were taken with a Leica SP5 and the Leica imaging software. Each channel was recorded independently and the z-movement increment was 150 nm. Further image processing was done with the ImageJ software and the ImageJ 3D Viewer .

Biocytin-filled L2/3 pyramidal cells were detected with a streptavidin-Cy3 antibody (1:500; Dianova, Hamburg, Germany), after an incubation period of 90 minutes at room temperature. NG2^-/-^ OPC were visible due to the expression of EYFP (where both NG2 alleles are replaced by EYFP). Immunostainings (n > 6 slices, from 4 animals) were examined by using a TCS SP5 confocal laser-scanning system (Leica Microsystems, Wetzlar, Germany) equipped with a Leica DM6000 CFS microscope, Ar/ HeNe continuous wave lasers and a 20x objective (NA 0.8). Colocalizations were evaluated from z-stack images (0.5 µm apart) with Leica Software (LAS AF; Leica Microsystems).

*Behavioral Tests*

All behavioral tests were performed with the same cohort of mice between 5-8 weeks of age. Only male littermates were used (11 NG2^-/-^ and 10 WT animals).

*Pre-pulse inhibition of acoustic startle response*

Separate cohorts of male and female mice (6-7 weeks old) was used for this experiment. Mice were group-housed in standard mouse cages with 12/12h light-dark cycle and *ad libitum* access to food and water. 11 NG2^-/-^ and 10 WT littermate mice were tested. The test was performed as described . Briefly, mice were placed in small metal cages (90 × 40 × 40 mm) to restrict major movements. The cages were equipped with a movable platform floor attached to a sensor that records vertical movements of the floor. The cages were placed in four sound-attenuating isolation cabinets (Med Associates, St.Albans, VT, USA). Startle reflexes were evoked by acoustic stimuli delivered from a loudspeaker that was suspended above the cage and connected to an acoustic generator. The startle reaction to an acoustic stimulus, which evokes a movement of the platform and a transient force resulting from this movement of the platform, was recorded with a computer during a recording window of 380 milliseconds (beginning with the onset of pre-pulse) and stored for further evaluation. An experimental session consisted of a 2-min habituation to 65 dB background white noise (continuous throughout the session), followed by a baseline recording for 1 min. After baseline recording, 8 pulse-alone trials using startle stimuli of 120 dB intensity and 40 milliseconds duration were applied in order to decrease the influence of within-session habituation. These data were not included in the analysis of the pre-pulse inhibition. For tests of pre-pulse inhibition, the 120 dB/40 milliseconds startle pulse was applied either alone or preceded by a pre-pulse stimulus of 70, 75 or 80 dB intensity and 20 milliseconds duration. An interval of 100 milliseconds with background white noise was employed between each pre-pulse and pulse stimulus. 10 trials of each kind (in total 40 trials) were applied in a pseudorandom order with variable inter-trial interval ranging from 8 to 22 seconds. Amplitudes of the startle response were averaged for each individual animal, separately for each type of trial (i.e. stimulus alone or stimulus preceded by a pre-pulse f 3 intensity). Pre-pulse inhibition was calculated as the percentage of the startle response using the following formula: PPI (%) = 100 – [(startle amplitude after pre-pulse and pulse / startle amplitude after pulse only) × 100].

*Behavioral assessment of olfaction*

Olfactory habituation/dishabituation test: This test consists of sequential presentations of different odors: Water, vanilla, rum. Each odor (or water) is presented in three consecutive trials of 2 min per trial with 1 min inter-trial interval (Yang and Crawley 2009). The food odorants were dissolved in water. Each mouse was presented with the fresh odor sample (1 ml) on filter paper. Odor samples were presented such that the mice have no opportunity to touch/bite the sample (i.e. it is enclosed in a perforated small Petri dish). Odors were presented in the clean standard mouse cage. Prior to presentations animals were habituated for 30min for this cage. Cumulative sniffing time was manually recorded during each odor presentation. Sniffing is scored when the animal is orientated towards the dish with its nose 2 cm or closer to the dish.

*Rotarod*

Rotarod is a test for motor function, balance and coordination and comprises a rotating drum which is accelerated from 4 to 40 revolutions per minute over the course of 5 min. Mice were placed individually on the revolving drum (Ugo Basile, Comerio, Italy). Once they had gained balance, the drum was accelerated. The time in seconds at which each animal fell the drum was recorded using a stop-watch. Each animal received three consecutive trials, the longest time on the drum being used for analysis.

*Computer-assisted method for gait analysis “CatWalk”*

Mice were subjected to gait assessment using the “CatWalk” automated gait analysis system (Noldus Information Technology, Wageningen, The Netherlands). The apparatus is made of a 1.3-m-long glass plate with dim fluorescent light beamed into the glass from the side. In a darkened environment (below 1 lx of illumination), the light is reflected downward and images of the footprints are recorded by the camera under the walkway when the animal’s paws come in contact with the glass surface. Mice were subjected to 3 consecutive runs of gait assessment. The images from each trial were converted into digital signals and processed and stored on a PC by “CatWalk” software. Following the identification and labeling of each footprint, a wide range of gait data was generated, as described .

*Contextual fear conditioning and extinction of fear conditioning*

These experiments were performed using a computerized fear conditioning system. Training took place in an apparatus consisting of a box (58 cm x 30 cm x 27 cm) with a simple grey interior and a 12-V light attached to the ceiling. The conditioning context consisted of a Plexiglas chamber (36 cm x 20 cm x 20 cm) placed on a removable shock grid made of stainless steel rods (4 mm in diameter, spaced 6 mm apart). The shock grid was connected to a shocker-scrambler unit delivering shocks of defined duration and intensity. For contextual conditioning, mice were trained with the following protocol: the pre-exposure time of 2 min in the conditioning box was followed by delivery of the shock (0.4 mA, 2 s) was delivered, and after 15 s the shock was delivered 2^nd^ time. During the pre-trial period, freezing behavior was monitored. The contextual memory test was performed 24 h after training. Mice were monitored for freezing for 2 min in the same context (chamber and interior) as used for training. Freezing behavior, defined as the absolute lack of movement (excluding respiratory movements), was recorded by video camera and analyzed by a computer. The data were converted to the percentage of time animals stayed frozen. For extinction of conditioned fear mice were repeatedly (4 times, once daily) exposed to the training context for 15 min and their freezing reaction was monitored for the first 2 min.

*Morris water maze*

Spatial learning and memory was assessed in a water maze . A large circular tank (diameter 1.2 m, depth 0.4 m) was filled with opaque water (25± 1˚C, depth 0.3 m) and the escape platform (10x10 cm) was submerged 1 cm below the surface. The swim patterns were monitored by the PC-based video-tracking system “Ethovision” (Noldus Inc., Netherland). The escape latency, swim speed, path length, and trajectory of swimming were recorded for each mouse. During the first two days mice were trained to swim to a clearly visible platform (“visible platform” task) that was marked with 15 cm high black flag mounted on it and placed pseudo-randomly in different locations across trials (non-spatial training). The extra-maze cues were hidden during these trials. After two days of “visible platform” training, “hidden platform” training (spatial training) was performed. For 10 days mice were trained to find a hidden platform (the flag was removed) that was located at the center of one of the four quadrants of the pool. The location of the platform was fixed throughout testing. Mice had to navigate using extra-maze cues that were placed on the walls. Every day they received four trials with an intertrial interval of 5 min. The mice were placed into the pool facing the side wall randomly at one of four start locations and allowed to swim until they found the platform, or for a maximum of 90 s. Any mouse that failed to find the platform within 90 s was guided to the platform. The animal then remained on the platform for 15 s before being removed from the pool. The next day after completion of the “hidden platform” training, a probe trial was conducted in order to determine whether the mouse uses spatial strategy to find the platform or not. The platform was removed from the pool and the mice were allowed to swim freely for 90 s. The time spent in the each quadrant of the pool was recorded.

*Statistics*

Statistical analysis was done by using SPSS or MS Exel. Data were tested for normal distribution with non-parametric one-sample Kolmogorov-Smirnov test. All quantitative data are expressed as mean ± SEM. Significance was classified as follows: *, p ≤ 0.05; **, p < 0.01; ***, p < 0.001; n.s. p > 0.05. Classified by Student’s *t* test, ANNOVA with "Dunnett's Multiple Comparison Test, non-parametric Wilcoxon–Mann–Whitney test or Bonferroni posttest.

**Supplemental References**

1. Jung M, Kramer E, Grzenkowski M, Tang K, Blakemore W, et al. (1995) Lines of murine oligodendroglial precursor cells immortalized by an activated neu tyrosine kinase show distinct degrees of interaction with axons in vitro and in vivo. The European journal of neuroscience 7: 1245-1265.

2. Valeva A, Walev I, Weis S, Boukhallouk F, Wassenaar TM, et al. (2004) A cellular metalloproteinase activates Vibrio cholerae pro-cytolysin. The Journal of biological chemistry 279: 25143-25148.

3. Biname F, Sakry D, Dimou L, Jolivel V, Trotter J (2013) NG2 Regulates Directional Migration of Oligodendrocyte Precursor Cells via Rho GTPases and Polarity Complex Proteins. The Journal of neuroscience : the official journal of the Society for Neuroscience 33: 10858-10874.

4. Diers-Fenger M, Kirchhoff F, Kettenmann H, Levine JM, Trotter J (2001) AN2/NG2 protein-expressing glial progenitor cells in the murine CNS: isolation, differentiation, and association with radial glia. Glia 34: 213-228.

5. Ludwig A, Hundhausen C, Lambert MH, Broadway N, Andrews RC, et al. (2005) Metalloproteinase inhibitors for the disintegrin-like metalloproteinases ADAM10 and ADAM17 that differentially block constitutive and phorbol ester-inducible shedding of cell surface molecules. Combinatorial chemistry & high throughput screening 8: 161-171.

6. Niehaus A, Stegmuller J, Diers-Fenger M, Trotter J (1999) Cell-surface glycoprotein of oligodendrocyte progenitors involved in migration. The Journal of neuroscience : the official journal of the Society for Neuroscience 19: 4948-4961.

7. Stegmuller J, Schneider S, Hellwig A, Garwood J, Trotter J (2002) AN2, the mouse homologue of NG2, is a surface antigen on glial precursor cells implicated in control of cell migration. Journal of neurocytology 31: 497-505.

8. Buxbaum JD, Thinakaran G, Koliatsos V, O'Callahan J, Slunt HH, et al. (1998) Alzheimer amyloid protein precursor in the rat hippocampus: transport and processing through the perforant path. The Journal of neuroscience : the official journal of the Society for Neuroscience 18: 9629-9637.

9. Karram K, Goebbels S, Schwab M, Jennissen K, Seifert G, et al. (2008) NG2-expressing cells in the nervous system revealed by the NG2-EYFP-knockin mouse. Genesis 46: 743-757.

10. Levenson JM, O'Riordan KJ, Brown KD, Trinh MA, Molfese DL, et al. (2004) Regulation of histone acetylation during memory formation in the hippocampus. The Journal of biological chemistry 279: 40545-40559.

11. Deepa SS, Carulli D, Galtrey C, Rhodes K, Fukuda J, et al. (2006) Composition of perineuronal net extracellular matrix in rat brain: a different disaccharide composition for the net-associated proteoglycans. The Journal of biological chemistry 281: 17789-17800.

12. Noh KM, Yokota H, Mashiko T, Castillo PE, Zukin RS, et al. (2005) Blockade of calcium-permeable AMPA receptors protects hippocampal neurons against global ischemia-induced death. Proceedings of the National Academy of Sciences of the United States of America 102: 12230-12235.

13. Schmid B, Schindelin J, Cardona A, Longair M, Heisenberg M (2010) A high-level 3D visualization API for Java and ImageJ. BMC bioinformatics 11: 274.

14. Neumann M, Wang Y, Kim S, Hong SM, Jeng L, et al. (2009) Assessing gait impairment following experimental traumatic brain injury in mice. Journal of neuroscience methods 176: 34-44.

15. Radyushkin K, Hammerschmidt K, Boretius S, Varoqueaux F, El-Kordi A, et al. (2009) Neuroligin-3-deficient mice: model of a monogenic heritable form of autism with an olfactory deficit. Genes, brain, and behavior 8: 416-425.

16. Morris R (1984) Developments of a water-maze procedure for studying spatial learning in the rat. J Neurosci Methods 11: 47-60.
